# Convergent evolution of polyploid genomes from across the eukaryotic tree of life

**DOI:** 10.1093/g3journal/jkac094

**Published:** 2022-04-22

**Authors:** Yue Hao, Jonathon Fleming, Joanna Petterson, Eric Lyons, Patrick P Edger, J Chris Pires, Jeffrey L Thorne, Gavin C Conant

**Affiliations:** 1 Biodesign Center for Mechanisms of Evolution, Arizona State University, Tempe, AZ 85281, USA; 2 Bioinformatics Research Center, North Carolina State University, Raleigh, NC 27695, USA; 3 Department of Biomedical Engineering, North Carolina State University, Raleigh, NC 27695, USA; 4 School of Plant Sciences, University of Arizona, Tucson, AZ 85721, USA; 5 Department of Horticulture, Michigan State University, East Lansing, MI 48824, USA; 6 Ecology, Evolutionary Biology and Behavior, Michigan State University, East Lansing, MI 48824, USA; 7 International Plant Science Center, New York Botanical Garden, Bronx, NY 10458, USA; 8 Division of Biological Sciences, University of Missouri, Columbia, MO 65211, USA; 9 Bond Life Sciences Center, University of Missouri, Columbia, MO 65211, USA; 10 Program in Genetics, North Carolina State University, Raleigh, NC 27695, USA; 11 Department of Statistics, North Carolina State University, Raleigh, NC 27695, USA; 12 Department of Biological Sciences, North Carolina State University, Raleigh, NC 27695, USA

**Keywords:** polyploidy, convergent evolution, reciprocal gene loss, evolutionary model

## Abstract

By modeling the homoeologous gene losses that occurred in 50 genomes deriving from ten distinct polyploidy events, we show that the evolutionary forces acting on polyploids are remarkably similar, regardless of whether they occur in flowering plants, ciliates, fishes, or yeasts. We show that many of the events show a relative rate of duplicate gene loss before the first postpolyploidy speciation that is significantly higher than in later phases of their evolution. The relatively weak selective constraint experienced by the single-copy genes these losses produced leads us to suggest that most of the purely selectively neutral duplicate gene losses occur in the immediate postpolyploid period. Nearly all of the events show strong evidence of biases in the duplicate losses, consistent with them being allopolyploidies, with 2 distinct progenitors contributing to the modern species. We also find ongoing and extensive reciprocal gene losses (alternative losses of duplicated ancestral genes) between these genomes. With the exception of a handful of closely related taxa, all of these polyploid organisms are separated from each other by tens to thousands of reciprocal gene losses. As a result, it is very unlikely that viable diploid hybrid species could form between these taxa, since matings between such hybrids would tend to produce offspring lacking essential genes. It is, therefore, possible that the relatively high frequency of recurrent polyploidies in some lineages may be due to the ability of new polyploidies to bypass reciprocal gene loss barriers.

## Introduction 

That organisms with doubled genomes existed was evident early in the history of genetics (Kuwada 1911; Clausen and Goodspeed 1925), and a lively debate was entered as to the implications of this fact. Wagner (1970) declared polyploidy to be “evolutionary noise” the same year that Susumu Ohno (1970) was giving it pride of place among the forces generating evolutionary innovations. The advent of genome sequencing changed the ground of this debate, opening new horizons of time for studies of the prevalence and influence of polyploidy. We know now that great branches of the eukaryotic evolutionary tree, including the vertebrates, all flowering plants and many yeasts, descend from ancient polyploids (Van de Peer *et al.* 2017), events that were difficult or impossible to detect with older data. For reasons that are not yet fully understood, many of these groups also show recurrent polyploidies, especially flowering plants (Soltis *et al.* 2009) and teleost fishes (Braasch and Postlethwait 2012).

With this extensive new set of polyploidies as a resource, other old questions can also be revisited, such as the relative prevalence of auto- and allopolyploids (Stebbins 1947). Allopolyploidy refers to hybridizations between distinct species that result in doubled (or more) genomes, while autopolyploids are derived from a single progenitor species (Kuwada 1911; Clausen and Goodspeed 1925; Stebbins 1947). Analyses of several paleopolyploid genomes have shown that while gene losses are common after polyploidy, in many cases the losses are not experienced equally by the two parental subgenomes (Thomas *et al.* 2006; Emery *et al.* 2018), a pattern known as biased fractionation. These biases are plausible but not definitive indicators of allopolyploidy.

There has also been controversy as to whether and how polyploidy affects the rate of speciation. Werth and Windham (1991) proposed that reciprocal loss of expression at duplicated loci could create Bateson–Dobzhansky–Muller incompatibilities between populations (see Orr 1996 for a history of this concept), because matings between them would give rise to offspring that did not express either copy of the gene. Reciprocal gene losses (RGLs) after polyploidy are an example of this process, and, were those genes essential, the offspring lacking their presence or expression would be inviable (Werth and Windham 1991) Such incompatibilities have been observed both in the wild and the laboratory (Mizuta *et al.* 2010; Maclean and Greig 2011). Muir and Hahn (2015) emphasize that RGL requires a period of reproductive isolation to form.

In the case of the ancient polyploidy in bakers’ yeast and its relatives, RGLs are commonly found between the descendant genomes, suggesting the potential for polyploidy to create new species by purely neutral means (Scannell *et al.* 2006, 2007). However, direct analyses of the speciation and extinction rates of lineages with and without recent polyploidy events has yielded inconclusive results, with some studies claiming reduced net diversification rates among polyploids and others disagreeing (Mayrose *et al.* 2011; Soltis *et al.* 2014a). More generally, the immediate and long-term adaptive value of polyploidy remains unclear: for instance, allopolyploids combine hybridizations with genome doubling and may derive immediate advantages from the hybridization effects rather than the doubling itself (Soltis *et al.* 2014b). Increased stress tolerance in polyploid organisms due to a variety of immediate and evolutionary mechanisms (Van de Peer *et al.* 2021) has also been invoked to argue for a radiation of polyploidy coincident with global catastrophes such as the KT mass extinction (Fawcett *et al.* 2009).

Hence, while many studies of the resolution of individual polyploidies have been made (Maere *et al.* 2005; Scannell *et al.* 2006; Thomas *et al.* 2006; Buggs *et al.* 2009a; Woodhouse *et al.* 2010; Braasch and Postlethwait 2012) and a few comparisons of several events are available (Paterson *et al.* 2006; De Smet *et al.* 2013; Garsmeur *et al.* 2014; Emery *et al.* 2018), no deep, cross-kingdom analyses of the patterns of postpolyploidy evolution using uniform and rigorous models have been undertaken. In the same vein, the similarities in which types of homoeologous genes are retained and lost after polyploidy (Seoighe and Wolfe 1999; Blanc and Wolfe 2004; Paterson *et al.* 2006; Freeling 2009; De Smet *et al.* 2013), as well as the prevalence of biased fractionation (Thomas *et al.* 2006; Garsmeur *et al.* 2014; Emery *et al.* 2018) are examples of pattern-based convergent evolution (Stayton 2015). However, a broad phylogenomic analysis of polyploidy with such uniform models is needed to ground this qualitative description of convergence with estimates of how similar or different the model parameters describing duplicate retention or biased fractionation are across these polyploidy events.

**Fig. 1.**
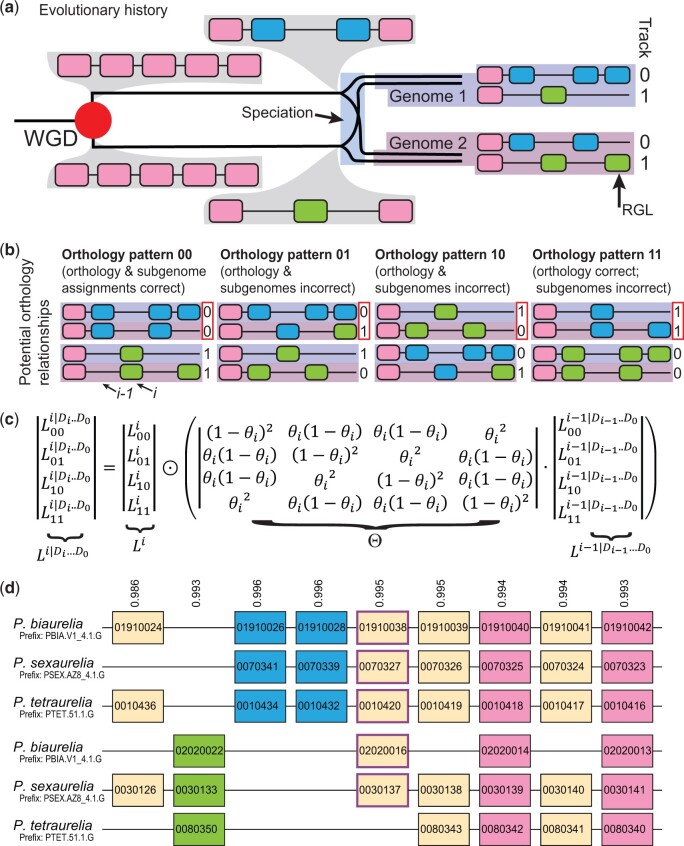
Inferring orthologous chromosome regions between polyploid genomes with POInT. a) Cartoon of gene losses and a speciation event after a whole-genome duplication. Immediately after the WGD, all five genes are present in 2 homoeologous copies. Three homoeologous gene losses occur before the split of the 2 species, 1 in the less fractionated subgenome (Track “0”; yielding the green gene present only in the lower window) and 2 from the more fractionated subgenome (Track “1”; yielding the 2 blue genes present only in the upper window). After the speciation event, Genome 1 loses a homoeolog from the more fractionated subgenome and Genome 2 loses one from the less fractionated subgenome, a case of reciprocal gene loss (RGL). b) There are 2^*n*^ = 2^2^ = 4 potential ways of phasing the chromosomal regions from Genome 1 relative to Genome 2 (i.e. of assigning orthology between the 2 regions). We identify these 4 states with the subgenome assignment for the top track for each of the 2 genomes (00→11; red boxed numbers at the right of each diagram). POInT uses a model of homoeolog loss to compute the likelihood of the observed gene presence/absence data at each locus (or “pillar”) for each of these 2^*n*^ relationships. These relationships each constitute a hidden state of the HMM implemented by POInT whereas a likelihood of observed gene presence/absence data for a relationship represents an emission probability for the HMM. c) Recurrence equation for computing the likelihood of each orthology assignment at pillar *i* conditional on the data at pillars 0 through *i-*1 (see b). For pillar *i*, we define a vector *L^i^* to be the likelihood of the orthology states, with elements *L*_00_^*i*^, *L*_01_^*i*^, *L*_10_^*i*^, and *L*_11_^*i*^ being POInT’s estimates of the likelihood of each such state based on the gene presence/absence data at that pillar. We then use a transition probability matrix, with each entry representing the probability that pillar *i* has a particular orthology state conditional upon another orthology state at *i-*1. The probability that the orthology state is maintained between pillars *i-*1 and *i* is 1-θ_*i*_ for each genome [and (1-θ_*i*_)^2^ in total]; the chance that one genome changes orthology state is θ_*i*_(1-θ_*i*_) and the chance that both change is θ_*i*_^2^. Here, θ*_i_* = θ, a global constant estimated from the data by maximum likelihood, except when synteny is not maintained between pillars, in which case θ_*i*_ = 0.5 (i.e. adjacent pillars do not inform on each other’s orthology state; see *Methods*). To compute a likelihood for the entire data set, POInT implements an HMM forward algorithm that expresses $L^{i|D_{i}\ldots D_{0}}$, the probabilities of orthology relationships for pillar *i* and the observed data at pillars 0 through *i* (denoted $D_{i}\ldots D_{0}$), in terms of the emission probabilities *L^i^*, the transition probabilities and the probabilities $L^{i-1|D_{i-1}\ldots D_{0}}$ that were already computed for pillar *i-*1*.* The vector of $L^{i|D_{i}\ldots D_{0}}\;$is then the element-wise vector product (indicated with the “⊙”) of $\Theta \cdot L^{i-1|D_{i-1}\ldots D_{0}}$ and *L^i^*. This formula can be applied sequentially starting at pillar 0, with the base case $L^{0|D_{0}}=\;L^{0}$. For *m* pillars, the overall likelihood of the dataset is then the sum of the elements of $L^{m-1|D_{m-1}\ldots D_{0}}.$ d) POInT employs posterior decoding to infer the orthology relationships at each pillar. Here, we illustrate a small region from the most recent *Paramecium* WGD (after phasing from the earlier duplication, see *Methods*), showing the set of orthology relationships inferred by posterior decoding. Genes in adjacent pillars that are also neighbors in an extant genome are shown connected by lines. The number above each pillar is the posterior probability of the inferred orthology relationship. The upper set of three tracks correspond to the less-fractionated parental subgenome, the lower three to the more fractionated one. Genes retained from *only* the upper less-fractionated genome are colored blue, from *only* the lower more fractionated one green, and fully retained duplicates are shown in pink. All other patterns of duplicate retention are shown in beige.

**Fig. 2.**
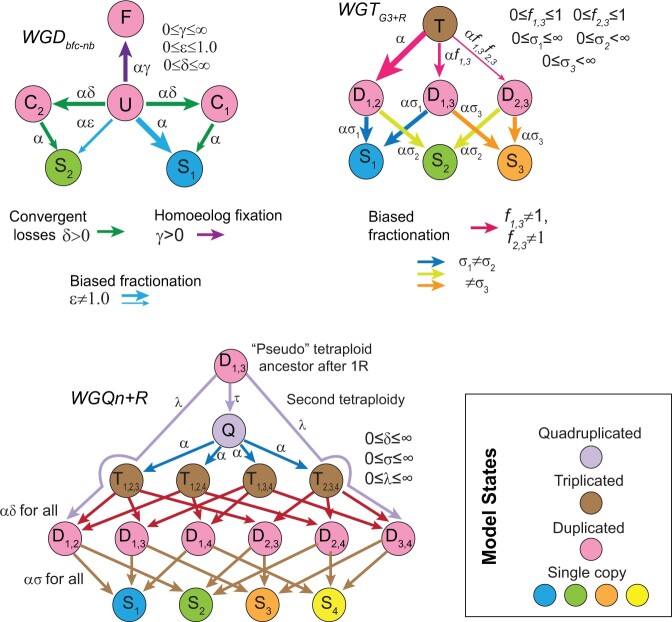
Models of polyploidy resolution for three types of events: WGD or whole-genome duplication/tetraploidy, WGT or whole-genome triplication/hexaploidy and WGQ or whole-genome quadruplication/octoploidy). **WGD:** all pillars start in state **U** (Undifferentiated), from which they can transition to either the three other duplicated states, **C_1_**(Converging state 1), **C_2_**(Converging state 2) and **F** (Fixed) or to the 2 single-copy states **S_1_**(Single-copy 1) and **S_2_** (Single-copy 2). **C_1_** and **S_1_** are states where the gene from the less-fractionated parental subgenome will be or are preserved, and **C_2_** and **S_2_** the corresponding states for the more-fractionated parental subgenome. The null model has parameters γ=δ=0 and ε=1.0. Homoeolog pair fixation is inferred when γ≠0, convergent losses when δ≠0 and biased fractionation when ε<1.0. **WGT:** in the base model all pillars start in state **T** (Triplicated) and transition first to duplicated states (***D_x, y_***) and hence to the single-copy states (***S_x_***). Genome 1 is assumed to be favored (fewer losses) and the identity of that genome inferred in the POInT computation. Losses from the triplicated state are then increasingly disfavored first to ***D*_1,3_** (parameter *f*_1__,__3_) and then to ***D*_2,3_** (parameter *f*_2__,__3_). There are also individual rates of loss from the duplicated to single-copy states (σ_x_). In the null model, *f*_1,3_*= f*_2,3_ = 1.0 and σ_1_= σ_2_= σ_3_. **WGQ:** Models of octoploid formation. The null model simply treats the four subgenomes as equivalent and as starting in the quadruplicated state (***Q***). This model has different loss rates from triplicated to duplicated loci (***T***_*x, y, z*_ to ***D_x, y_*,** parameter δ) and from duplicated to single-copy loci (***D_x, y_*** to ***S_x_*,** parameter σ). A formation model for the octoploidy can then be added: all pillars start in state ***D*_1,3_** and can symmetrically experience a gene loss from genome 1 or 3 (parameter λ) and transition to state ***D*_1,2_** or ***D*_3,4_** or become quadruplicated (null transition). The three models illustrated here are the most complex model fit to the various events, including the parameters associated and their numerical ranges.

Using our tool for modeling the evolution of polyploid genomes, POInT (the Polyploidy Orthology Inference Tool; Conant and Wolfe 2008), we explored the resolution of ten independent polyploidies. We adopt the term “homoeolog” below to refer to homologous genes produced by any type of polyploidy rather than “duplicate” or “ohnolog” because the events considered comprise several distinct types of polyploidy. The hallmark of polyploidy in a genome is a pattern of interleaved synteny, comprising not just the surviving homoeologs but also single-copy genes that are now found in interleaved positions on pairs (or more) of chromosomal segments homologous to the ancestral single-copy regions. In Fig. 1a, we show an example of this evolutionary process, which yields conserved synteny blocks in the extant genomes. Those synteny blocks differ between genomes, meaning it is necessary to “phase” them into orthologous regions. As shown in Fig. 1b, for a set of *n* tetraploid genomes, there are 2^*n*^ possible orthology relationships at each ancestral locus. We use the term “pillar” to denote all of the genes or lost homoeologs at such a locus. POInT computes the likelihood of the observed homoeolog presence/absence data at each pillar for each possible orthology relationship. Via a hidden Markov model (HMM) that combines the possible orthology relationships for each pillar with the syntenic organization among pillars (Fig. 1c), POInT employs posterior decoding to infer orthology estimates for each pillar with associated posterior probabilities (top of Fig. 1d) as well as estimates of the model parameters describing the process of homoeolog loss (Fig. 2).

**Fig. 3.**
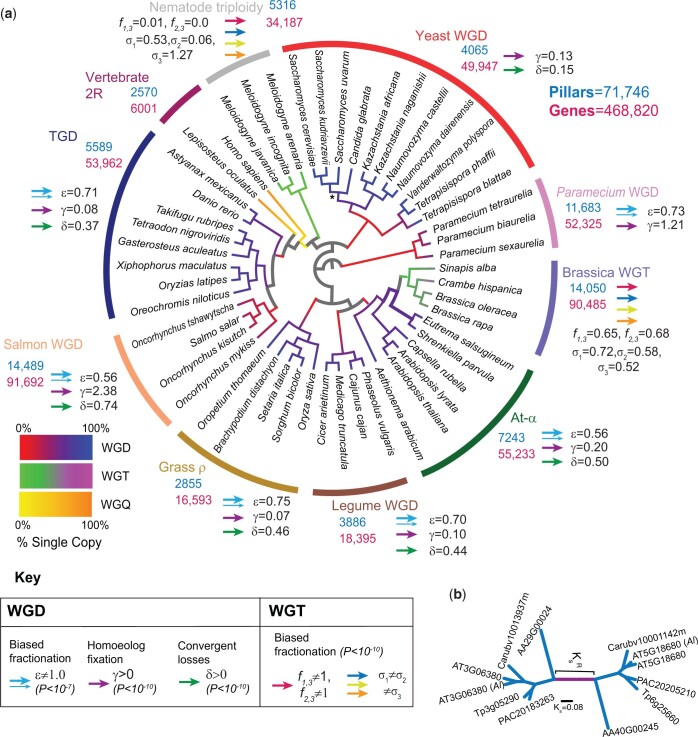
a) The assumed or computed phylogenetic relationships among species sharing the ten polyploidies studied (see *Methods*). Gray branches are those where no polyploidy event was studied. Because the temporal divergences of various groups are not well established, the tree is illustrated in an ultrametric format (Scaled topologies are shown in Supplementary Fig. 1). Each polyploid branch is colored using POInT’s estimates of the proportion of loci that were single-copy at its beginning and ending. Corresponding color keys for WGD, WGT, and WGQ events are shown. The number of “pillars” (homoeologous loci) and the total number of gene models studied across each event are noted, as are the total number of loci and genes considered. The “*” on the yeast WGD branch indicates the branch where the proportion of genes returned to single-copy that are presently essential was tested (Supplementary Table 8). Next to each event, we show arrows and parameter estimates indicating postpolyploidy evolutionary processes such as biased fractionation for which we found significant evidence in that event (see key). b) An example mirrored gene tree for a completely retained set of homoeologs from At-α, illustrating the trees from which synonymous divergences were estimated. The branch lengths are given in number of synonymous substitution per synonymous site (i.e. *K_s_*), with the shared internal (i.e. “root”) branch shown in purple (*K_s_^R^*). For analysis purposes, the length of this branch was always divided by 2 to be comparable to the remaining branches (i.e. split at its midpoint).

Our analyses here encompass a total of 50 polyploid genomes and more than 460,000 individual genes (Fig. 3). We find that the patterns of gene loss after these different events show strikingly similar patterns, with strong evidence for biased fractionation and homoeolog fixation. Using synonymous substitutions as an evolutionary clock, we show that the rate of gene loss immediately after polyploidy is generally higher than in later periods. RGL is also prevalent after all of these polyploidy events, and we suggest it might introduce barriers to hybridization that could be overcome through subsequent allopolyploidy events.

## Methods

### Synteny block inference

Our three-step pipeline for inferring blocks of pillars with *n-*fold conserved synteny (NCS) produced by polyploidy (Conant 2020) first uses GenomeHistory (Conant and Wagner 2002) to find all pairs of homologous genes between each polyploid genome and an outgroup lacking the event in question (see Supplementary Table 1 for genome details and Supplementary Table 2 for parameters). The second step seeks to place these homologous genes into *N* : 1 relationships between the polyploid genome and the outgroup (*N = *2 for a WGD, *N = *3 for a hexaploidy and *N = *4 for an octoploidy). Using simulated annealing (Kirkpatrick *et al.* 1983), this step proposes sets of ordered pillars, each of which contains a single gene from the outgroup that lacks the polyploidy (*G*) and no more than *N* of the homologs of that gene from the polyploid genome. The annealing algorithm then seeks a combination of these assignments and a relative ordering of the *m* outgroup genes *G*_1_*.G_m_* that maximizes the number of synteny relations. We define 2 genes to be in synteny if they are neighbors in the genome, ignoring any genes without homologs to the compared genome. In the third step, these NCS blocks for each polyploid genome are merged across all of the polyploid genomes. In this merging, only pillars where we have at least one homologous and syntenic gene from each polyploid genome are included. With the set of merged pillars, a further simulated annealing search is undertaken to infer a global pillar order that minimizes the number of synteny breaks. While not strictly an ancestral genome inference (Sankoff and Blanchette 1998), it is helpful to think of this optimal ordering as approximating the order of the genes just prior to the polyploidy event. Our previous work has shown that this inference approach is highly specific, with no apparent cases of paralogous genes not created by the polyploidies in question being included in the pillars (Emery *et al.* 2018; Conant 2020).

### Modeling polyploidies with POInT

At each pillar, POInT calculates the probability of the observed gene presence-absence data conditional upon all possible orthology relationships and a phylogeny. It carries this uncertainty in orthology through its likelihood computations using a HMM that resembles the Lander-Green approach for constructing linkage maps on a pedigree (Lander and Green 1987). The parameter θ_*i*_ corresponds to the probability that the inferred orthology relationships change between syntenic neighbors at pillars *i-*1 and *i*. When a pair of pillars are separated by a synteny break (i.e. the 2 genes are not each other’s chromosomal neighbors), their orthology relationships are independent (i.e. θ_*i*_ = 1/2). Otherwise, θ*_i_* = θ, a global parameter estimated from the data by maximum likelihood.

This modeling framework allows for testing hypotheses about postpolyploidy gene losses. We have extensively validated it in several prior contributions (Conant and Wolfe 2008; Conant 2014; Emery *et al.* 2018; Conant 2020). For tetraploidies, we analyzed 3 phenomena: fixation of homoeolog pairs, biased fractionation and overly frequent parallel losses of the same homoeolog on independent branches of the phylogeny (Supplementary Table 3). For the *Brassica* hexaploidy and nematode triplication events, we focused on differences in homoeolog loss rates between the 3 subgenomes (Supplementary Table 4). We further allowed the root branch to have separate values of the model parameters to account for the 2-step nature of hexaploidy formation (Fig. 2; Tang *et al.* 2012).

### Analyzing nested genome duplications with POInT

The paramecia studied here and all vertebrates descend from 2 sequential genome doubling events (hence the “2R” events in vertebrates). As a result, these genomes have an octoploid state relative to the outgroup used. To model such a whole-genome quadruplication (WGQ), we first used a null model (WGQ_*n*_; Fig. 2) where losses occur equally from all four subgenomes, but where the loss rate from triplicated and duplicated loci can differ from that seen in quadruplicated loci. To model the 2-step formation of a WGQ, we assumed that the first WGD produced an intermediate polyploid genome where all pillars were in state *D*_1,3_. Before the second WGD, genes could be lost either from subgenome 1 or subgenome 3, such that, when the second WGD occurred, some pillars are quadruplicated, and some are in state *D*_1,3_, because they transitioned from *D*_1,3_ to *S*_1_ and then to *D*_1,2_ at the second event, and some are similarly in state *D*_3,4_ (Fig. 2).

These WGQ models present a challenge because the POInT computation for such an octoploidy with *n* genomes scales as *O(2*4^2^*^n^)*. As a result, it is only computationally feasible to analyze 2 octoploid genomes. However, if the consecutive whole-genome doublings were sufficiently separated in time, POInT can separate them using the 2-step model just described. To do so, we compute the posterior probabilities for each subgenome assignment at each pillar. We are interested in pairs of genomic regions that share a high probability of descending from the same original duplicated region created by the first WGD. This origin is marked by those regions having a high probability of belonging either to subgenomes 1 and 2 or to 3 and 4. We thus sought to phase regions from both octoploidies into pairs of regions created by the most recent genome doubling. For the ciliate genomes, we were able to phase the quadruplicated loci into 11,683 pairs of duplicated loci with at least one gene from each genome and where our orthology assignment confidence for assigning extant genes to one of the 2 subgenomes from the *first* polyploidy event was ≥99%. Our results are largely consistent with earlier analyses of these genomes, which also suggested that *Paramecium sexaurelia* branched first after the second event and that the event and its aftermath were marked by RGL and gene conversion (McGrath *et al.* 2014a, 2014b). However, those authors argued that the recent WGD was likely an autopolyploidy because they detected only modest biases in duplicate loss propensities between syntentic blocks (McGrath *et al.* 2014a). POInT’s global bias parameter applied to the larger dataset used here provides significant evidence for biased fractionation; it appears therefore that the recent *Paramecium* event may have been an allopolyploidy. For the vertebrate 2R events, a model that attempts to phase the 2R duplicates fit the data no better than did the null model (*P = *0.1, likelihood ratio test with 1 *d.f.*) and so no further phasing was attempted.

### Biased fractionation and convergent losses

The full WGD_bfc-nb_ model used for our main analyses includes convergent loss states C_1_ and C_2_. When we fit a model (WGD_bfc_) that allows a fractionation bias to also exist between these 2 states, we find that that model fits the data no better than the (unbiased) WGD_bfc-nb_ model (Supplementary Table 5). Hence, the *ε* parameters in Fig. 2 only reflect the degree of bias observed for pillars passing directly from state U to S_1_ or S_2_. However, the conclusion of the presence of biased fractionation in these genomes is still strongly supported when models without convergent losses are used (Supplementary Table 5), even if, in some cases, the *ε* estimates are somewhat higher for those models.

### POInT and topological inference

For the legume WGD, the grass ρ event, the Paramecium tetraploidy, the nematode triploidy and the salmonid WGD, we used POInT to infer the maximum likelihood phylogeny under the WGD_bfc-nb_ or WGT_G3_ models and an exhaustive tree search (Supplementary Fig. 1). For the Brassica WGT, we assumed that *Brassica* *rapa* and *Brassica* *oleracea* were sister taxa and tested all three rooted topologies consistent with this constraint. The topology for the yeast WGD was taken from Kurtzman and Robnett (2003), for the TGD from Near *et al.* (2012), and for At-α from Huang *et al.* (2016). The vertebrate 2R topology is trivial.

For the salmonid WGD, the inferred topology differs significantly from others that have been published. We, therefore, fit the full POInT model under the topology published by Crespi and Fulton (2004). The orthology estimates and model parameters are largely unaffected by this topology change: the orthology relationships of only 106 (0.7%) pillars with posterior probability >80% differ when the topology is changed, and 91 of these changes simply swap the identities of the more and less fractionated genomes. The corresponding figures for 95% confidence are 9 and 7 pillars.

### Orthology inferences and inference of synonymous distances

Using high confidence orthologs estimated with POInT, we computed the mean synonymous divergence for every branch for each polyploidy event. The nematode triploidy and vertebrate 2R events were omitted from this analysis due to their fragmented synteny blocks. For the tetraploidies, we considered “nearly fully duplicated” pillars: i.e. pillars with at most one missing gene copy from each of the 2 gene trees produced by the genome duplication (2 total losses) for all events except the TGD and yeast WGDs, where we allowed 2 losses from each subtree (4 total losses). For the Brassica hexaploidy, we analyzed only fully triplicated pillars. At each such pillar, we aligned amino acid sequences for the genes in question with T-coffee (Notredame *et al.* 2000). We fit the Goldman and Yang codon model of evolution (Goldman and Yang 1994) to the corresponding codon-preserving alignments and mirrored gene trees and extracted the estimated synonymous divergence (*K_s_*) for each branch from this codon model as described by these authors.

With the possible exception of the salmonids and ciliates (Allendorf and Thorgaard 1984; Macready *et al.* 1996; Braasch and Postlethwait 2012), all of the events studied here are believed to be allopolyploids (Thomas *et al.* 2006; Schnable *et al.* 2012; Tang *et al.* 2012; Marcet-Houben and Gabaldon 2015; Conant 2020; Schoonmaker *et al.* 2020). For a given pillar in set of allopolyploid taxa, the mean synonymous divergence observed along this root branch ($\bar{K_{s}^{R}}$; Fig. 3) should represent the sum of the prepolyploidy divergence of the diploid progenitors as well as the divergence that occurred after the formation of polyploid but before the first speciation event among the polyploid taxa. However, recombination events could, through genetic drift, result in the replacement of alleles from one of the progenitors with those from the other (Wolfe 2001). These recombinations, or homoeologous exchanges (HE; Gaeta and Pires 2010) are reasonably common in neopolyploid plants (Doyle *et al.* 2008; Chalhoub *et al.* 2014; Zhang *et al.* 2020), but it is not clear whether they are frequent enough to affect the divergence seen along these root branches. Postpolyploidy homoeolog displacement (Gaut and Doebley 1997; Wolfe 2001) will erase the divergence between the progenitor genomes, leaving only the postdisplacement divergence to be observed. In such a case, we might expect to observe 2 modes in synonymous divergence, a larger value for homoeologs that did not experience displacement and a smaller one (lacking the progenitor divergence) for homoeologs that did. To test this hypothesis, we fit the set of estimated synonymous divergences (K_s_) along the root branches to either 1 or 2 log-normal distributions using the R package mclust (Scrucca *et al.* 2016) with the best-fit model (i.e. 1 or 2 distributions) chosen with the Bayesian information criterion (BIC; Schwarz 1978). Values of K_s_ less than 5 × 10^−3^ or greater than 2.0 were omitted from these analyses as representing either no synonymous divergence or saturated synonymous divergence, respectively. When 2 distributions were fit, a “weighting” *p* reflecting the mixing proportion of each component was also estimated. For a few root branches, a bimodal distribution is preferred. However, in most cases, this bimodality is not consistent across different collections of pillars and, even when it is, the proportion of pillars belonging to one of the “modes” is generally very small (Supplementary Table 6). We hence see little suggestion of HE in these data.

### Filtering for extreme instances of gene conversion

Because gene conversion among homoeologs (as seen in yeasts; Evangelisti and Conant 2010; Scienski *et al.* 2015) could confound our *K_s_* estimates, we sought to filter out pillars that showed strong evidence of having experienced it. We created “gene conversion gene trees” for each pillar where each homoeologous gene was forced to be sister to its paralog(s). Any pillars where the likelihood of the sequence alignment under these gene conversion trees was higher than that seen in the mirrored species trees was omitted from our estimates of synonymous divergence (Supplementary Fig. 2).

### Comparing duplicate loss rates to estimated synonymous divergence

Using the *K_s_* inferences made above for each branch, we compared POInT’s maximum likelihood estimate (MLE) of the rate of homoeolog loss (i.e. its estimated branch length, αt in Supplementary Fig. 1) to each branch’s mean synonymous divergence, $\bar{K_{s}}$, to see if the number of losses on any particular branch was unusually large or small. Previous studies that used gene tree approaches to inferring loss rates (Tiley *et al.* 2016) are not comparable to the results here because, unlike POInT, they do not account for the fact that the *observed* homoeolog loss rate necessarily declines in time because progressively fewer homoeolog pairs remain to be lost. Similarly, prior parsimony-based analyses do not include the uncertainty inherent in estimating loss timing, which we account for using POInT’s explicit phylogenetic models (McGrath *et al.* 2014a). Estimating confidence intervals for these ratios of $\alpha \cdot t/\bar{K_{s}}$ is challenging. We treated the numerators and denominators of these ratios as being normally distributed and independent random variables. The MLEs of αt in the numerators should have asymptotically normal distributions with means that are equal to the true parameter values. The variances of these normal distributions were approximated by evaluating the inverse of the observed Fisher information (i.e. the Hessian of the negative log-likelihood; see Kendall and Stuart 1973). We estimated the observed Fisher information values via a single-dimension finite difference approximation that ignored covariances between the αt parameter and other parameters.

For each branch of the phylogeny, the *K_s_* estimates that are in the denominator of the ratio $\alpha \cdot t/\bar{K_{s}}$ are obtained via a sample mean of the *K_s_* estimates from the sequences of individual pillars (i.e. $\bar{K_{s}}$). Due to the Central Limit Theorem, this sample mean should be approximately normally distributed with mean equal to the true parameter value and with variance being approximately the sample variance among individual *K_s_* estimates divided by the number of individual *K_s_* estimates.

To infer confidence intervals for the ratio of $\alpha \cdot t/\bar{K_{s}}$ on each branch, we independently sampled from the 2 aforementioned normal distributions that are used to approximate the uncertainty of αt and $\bar{K_{s}}$ estimates in the ratio. For each branch, we calculated the ratio of these sampled values for 1,000 pairs of randomly drawn values. We then sorted the resulting ratios and set 95% confidence intervals by finding the ratio value that defined the lower and upper 2.5% of the sorted values.

Because the inclusion of fixation in our loss models can give rise to long tip branches (effectively the model suggests that all surviving duplicates in some genomes are now fixed), we present data using a model with convergent losses and biased fractionation but no fixation (WGD_bc-nb_). However, our results are very similar when using the full WGD_bfc-nb_ model (Supplementary Fig. 3).

### Potential biases in estimating the rate of early duplicate losses

**Fig. 4.**
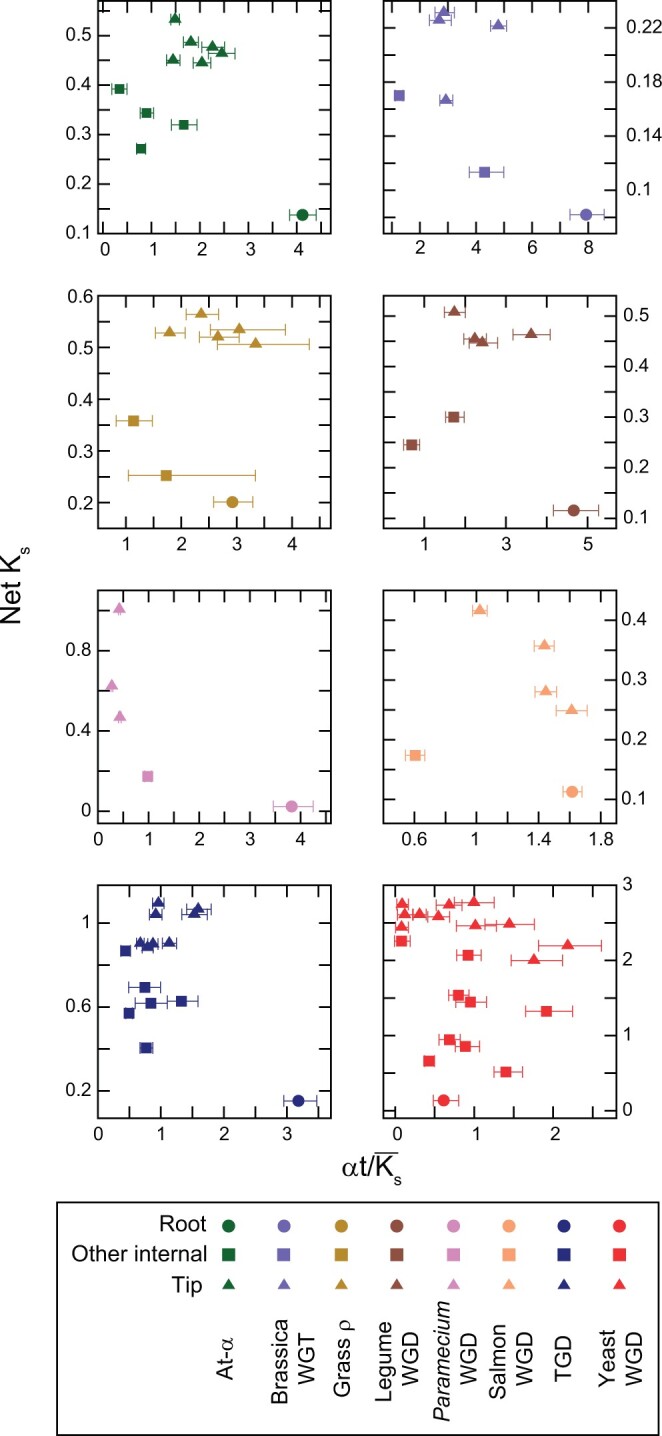
Rapid loss of homoeologs immediately after polyploidy. On the *x-*axis is the ratio of rate of homoeolog loss (the αt branch length estimate from POInT’s models, see Fig. 3) and the estimated mean synonymous divergence for that branch ($\bar{K_{s}}$; see *Methods*). Hence, larger values of this ratio indicate more homoeolog losses per unit K_s_. For the At-α, Brassica WGT, Legume WGD, Paramecium WGD and the TGD, the $\alpha \cdot t/\overset{-}{K_{s}}$ ratio for the root branch is significantly larger than seen on any other branch (c.f., the 95% confidence intervals shown, computed as described in the *Methods* section). For these panels, we used a model excluding duplicate fixation here because including fixation in the model occasionally results in very long estimates of tip branch lengths (see *Methods*). However, our conclusions are similar under the full WGD_bfc-nb_ model (see Supplementary Fig. 3). On the *y*-axis is the net synonymous divergence to the end of the branch in question: in other words, the sum of the synonymous divergence of that branch and all its ancestors back to the root branch. This net divergence value is a rough indicator of the time since the polyploidy event for each branch. The root branch is indicated with a circle, other internal branches with squares and tip branches with triangles.

One might object that this signal of rapid early duplicate losses might instead be due to genes being missing from one of the allopolyploid progenitors, meaning that the duplicate pair in question never formed. In this case, the estimates of loss rates along the root branch might be inflated. A priori, this idea appears unlikely because of the nature of the genes selected for analysis with POInT. Our inference pipeline requires that each pillar be mapped to a single-copy gene in an outgroup genome (Supplementary Table 1) and that every polyploid genome possess at least 1 copy of that gene. Hence, the genes analyzed are on average very well conserved over the tree, making a large number of losses of such genes in a progenitor unlikely. Furthermore, in the special case of a hexaploidy, we can actually use POInT to estimate the proportion of genes missing from at least one progenitor genome. Specifically, for the *Brassica* hexaploidy, we showed that the proportion of pillars where a gene was missing from the last-arriving progenitor subgenome (termed LF, or “least fractionated”) was only ∼0.3% (Hao *et al.* 2021). Finally, we can also explore the hypothesis of a large number of missing progenitor genes by looking at the patterns of biased fractionation on the root branch relative to the other branches of the tree. We fit a model where the biased fractionation parameter *ε* was allowed to differ on the root branch relative to the other branches, using the WGD_bf_ model above to avoid concerns with convergent losses. Losses from the progenitor genomes prior to polyploidy should be balanced, since biased fractionation is driven by forces that appear at the polyploidy event. Hence, under a model of numerous prepolyploidy losses, the fractionation bias on the root branch should be *lower* (larger *ε*) than on subsequent branches. Instead, in several cases, the level of biased fractionation is actually higher on the root branch (i.e. the inferred value of *ε* is smaller along the root branch for At-α and the paramecium and salmonid WGDs; Supplementary Table 5), consistent with our prior observations in yeast (Emery *et al.* 2018). Given this fact, and because in some cases upwards of 50% of the currently fully single-copy genes in these genomes were returned to single copy along this root branch (Supplementary Fig. 4), the degree of prepolyploidy losses that would be required to bias the results in Fig. 4 is implausibly high.

### Comparisons of selective constraint for different classes of polyploid loci

We examined the inferred average selective constraint (*K_a_*/*K_s_*, estimated as described above) for 5 classes of polyploid loci (i.e. pillars) across the seven WGD events: (1) Pillars that are single copy in all taxa and have a high probability of having returned to single-copy along the root branch, (2) Pillars that are completely single copy but where the genes did not return to single-copy on the root branch (i.e. where alternative copies of the duplicated genes are preserved in different genomes), (3) pillars with duplicates surviving in only a single species, (4) pillars where all but one species maintains the duplication, and (5) pillars where all species maintain duplicate copies. Confidence intervals for these mean *K_a_*/*K_s_* estimates were estimated with the approach described above.

### Identifying RGLs between polyploid taxa

**Fig. 5.**
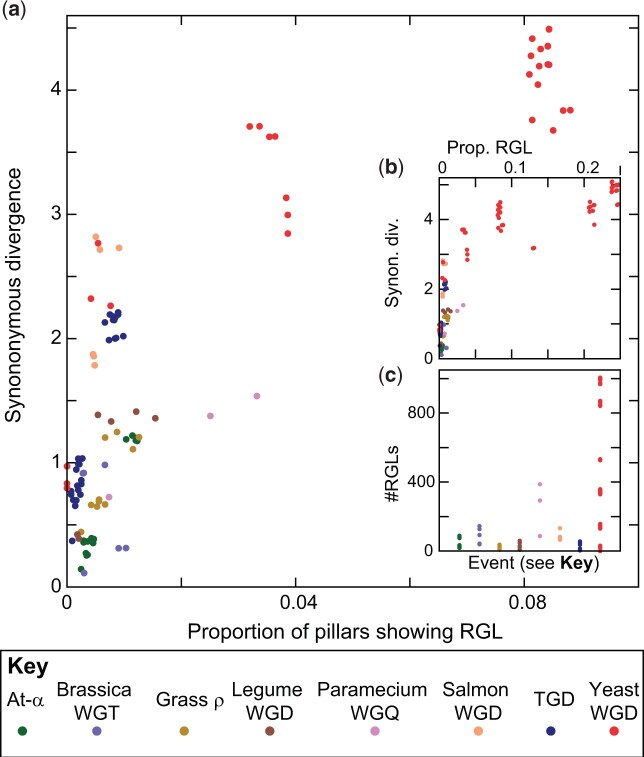
RGL after polyploidy. a) RGLs between pairs of polyploid taxa (*x*-axis, normalized by the total number of loci/pillars analyzed for that event) as a function of the inferred synonymous divergence of those taxa (*y*-axis). Panel (a) gives a cropped view that focuses on RGLs in the nonyeast taxa, while panel (b) (shows how the RGL frequencies in yeast dramatically exceed those for the remaining events. For each pair of taxa from a given event, we identified all single-copy loci in the 2 genomes where POInT infers a 95% or greater confidence that those genes are paralogs created by the ancient polyploidy and not more recent orthologs produced by the postpolyploidy speciation events. There are roughly linear relationships between RGL frequency and synonymous divergence. Because the data points shown are phylogenetically dependent (different species pairs share considerable common evolutionary history), we have not attempted to fit regression lines to these data. Standard approaches to phylogenetically independent contrasts (Felsenstein 1985) do not apply here as the inferred RGLs are pairwise species traits and not independent measures on each taxon. It is however notable that the asexually reproducing yeasts appear to accumulate more RGLs per unit *K_s_* than other taxa. b) As for (a) but including the full range of RGL prevalence in the taxa sharing the yeast WGD. c) Total numbers of RGLs inferred for each pair of taxa for each event (*x*-axis).

For a pair of single-copy genes from distinct genomes, the probability that these genes represent RGLs is simply the sum of the probabilities of the orthology relationships, estimated with POInT, that place them as paralogs rather than orthologs. We computed, for each pair of extant taxa in each event, the set of RGLs that we could identify with a confidence of ≥95% (Fig. 5a). To avoid spurious inferences, we restricted our identification of RGL pairs to single-copy genes in each genome where either: (a) both the gene and the “hole” corresponding to its lost homoeolog were in synteny with genes on either side or (b) the single-copy gene in question was the only homolog of the outgroup gene used for the inference of the NCS blocks. In the first case, this filter corresponds to a clear absence of a corresponding homoeolog in the paralogous synteny block, in the second to the absence of a gene that could be the “missing” homoeolog. We then used TBLASTX (Altschul *et al.* 1997) to search the noncoding regions of each genome for putative homoeologous copies of the inferred RGL gene that were missed in the genome annotations (i.e. the inference of RGL was spurious due to an annotation artifact). In Case “a” above, this search was restricted to the noncoding regions in the “hole” between the neighboring syntenic genes; in Case “b,” we searched the entire genome for the potentially unannotated homoeolog. Only RGL genes with no such matching noncoding regions at an *E*-value cutoff of ≤10^−10^ were considered “true” RGLs. These secondary filters were not applied for the yeast WGD because those data were taken from the manually curated Yeast Genome Order Browser (YGOB, Byrne and Wolfe 2005).

Data on gene knockouts producing lethal phenotypes from zebrafish, *Arabidopsis* *thaliana* and bakers’ yeast were taken from ZFIN (Howe *et al.* 2013; Conant 2020); a set of 510 “embryo-defective” genes identified by Meinke (2020); and Steinmetz *et al.* (2002), respectively. The proportion of RGLs in these “essential gene” lists was compared to the proportion of all other single-copy genes from the same organism in the list using Fisher’s exact test (Sokal and Rohlf 1995). For these same three species, we used GeneOntology data (Gene Ontology Consortium 2015) and Panther Overrepresentation Tests (Release 20200728; Mi *et al.* 2019) to ask if there were terms from the GO-Slim Biological Process, Cellular Compartment, or Molecular Function ontologies that differed in their frequency between the RGL genes and other single-copy genes. After FDR correction (Benjamini and Hochberg 1995), no such terms were found for any of the three ontologies across any of the 3 genomes (FDR-corrected *P-*value > 0.05).

## Results

### Modeling evolution after 10 independent polyploidies

Using POInT, we assembled a set of ∼70,000 homoeologous loci produced by 10 different polyploidies. For each polyploidy event, we inferred a set of pillars that it created and ordered them so as to maximize the retained synteny among the extant genes, approximating the ancestral order of the single-copy genes just prior to polyploidy (see *Methods*). Six of the events are whole-genome duplications (WGDs or tetraploidies): At-α in *A.* *thaliana* and its relatives, a WGD found in legumes, the ρ event from grasses, the teleost-specific genome duplication (TGD), and WGDs from salmonids and yeasts. We further analyzed an asexual triploidy in nematodes, a hexaploidy (whole-genome triplication; WGT) in cabbages and their relatives (*Brassica* WGT) and 2 octoploidies: the vertebrate 2R polyploidy and another in the paramecia (Fig. 3). Analyzing octoploidies in POInT is computationally expensive. As a result, we modeled the octoploidy among the paramecia as occurring via 2 sequential genome duplications and then extracted and analyzed only the more recent of these 2 events for the remainder of our work (see *Methods*). This approach failed with the vertebrate 2R event, presumably because the 2 events are very ancient and closely spaced in time. A visual interface to these data is available from the POInT browser (http://wgd.statgen.ncsu.edu; accessed 2022 Apr 28).

For the WGD events, we compared nested models of evolution (Fig. 2 and Supplementary Table 3) that describe the process of homoeolog loss after polyploidy: these models differ as to whether they include biased fractionation, homoeolog fixation and convergent homoeolog losses. For all seven tetraploidies, models that allow for homoeolog pairs to be retained as fixed duplicates after polyploidy fit the observed loss data better than models without such an effect (γ≠0; *P < *10^*−*10^; likelihood ratio test or LRT; Figs. 2 and 3). In addition, every event save that in yeast shows strong evidence for biased fractionation (ε≠1; *P < *10^*−*7^; LRT; Figs. 2 and 3), while all but the *Paramecium* event show a pattern of independent yet convergent losses to the same homoeolog in independent lineages (δ≠0; *P < *10^*−*10^; LRT; Figs. 2 and 3). The nematode triploidy and the *Brassica* WGT also share similar patterns of biased fractionation (Figs. 2 and 3 and Supplementary Table 4).

The fact that these events are of widely differing ages is evident from the different degrees of loss/resolution seen in the extant genomes. The branches of Fig. 3 are color-coded by POInT’s inferences of the proportion of single-copy genes (i.e. loci where all but one of the homoeologous genes have been lost) present at their beginning and ending. While the yeast WGD is inferred to be nearly “fully” resolved (nearly all homeologous loci have been reduced to single-copy or fixed as duplicates), the tetraploidy in salmonid fishes and the nematode triploidy show proportionally few single-copy genes. The nematode triploidy differs from the remaining events in that these animals are asexual triploids and are likely under a different selective regime in their gene losses, (Schoonmaker *et al.* 2020). The continued occurrence of meiotic chromosome pairings of homoeologous chromosomes created by the salmonid event may have reduced the rate of homoeolog loss in those genomes (Allendorf *et al.* 2015).

### Many events show rapid homoeolog loss immediately after polyploidy

Loss of duplicate genes immediately after polyploidy can be rapid (Scannell *et al.* 2006, 2007), and at least 2 nonexclusive hypotheses exist as to why. The first is that genetic drift should eliminate truly redundant gene copies quickly (Li 1980; Lynch and Conery 2000). The second is the potential for “selected” duplicate losses, an idea suggested by the observation of gene families found to be persistently returned to single-copy after independent polyploidies (Paterson *et al.* 2006). Such losses might occur if the increases in gene copy number after polyploidy induce disadvantageous dosage conflicts for these genes, such that natural selection acts to remove the homoeologous copies in question (Edger and Pires 2009; De Smet *et al.* 2013).

To study the pattern of early losses, we examined the divergence that occurred immediately after the polyploidy event and before any speciation events. In the context of a gene tree for a pair of homoeologous genes produced by a WGD, this period corresponds to the internal branch of the gene tree separating that pair of homoeologs. For a WGT, the situation is analogous except that there are three such branches separating the three homoeologous copies. For simplicity, we refer to these branch(es) as the “root” (purple in Fig. 3b). For all branches in each event, we obtained a rough estimate of the time encompassed by that branch by using the mean number of synonymous substitutions per synonymous site ($\bar{K_{s}}$) across many homoeologous genes as a neutral clock (see *Methods*). The rate of homoeolog loss for each branch is given by POInT’s branch length estimate (αt), computed with its irreversible loss model, such that these branch lengths are scaled based on the number of homoeologous copies at the beginning of that branch (meaning that they are not biased by the fact that later branches have fewer total homoeologs available for loss, see *Methods*). The ratio of $\alpha \cdot t/\bar{K_{s}}$ gives a sense of whether homoeolog losses per time are unusually high or low for a given branch relative to other branches in the same event. For the majority of the polyploidies, we found that the $\alpha \cdot t/\bar{K_{s}}$ ratio was higher for the root branch than any other branch, consistent with a more rapid loss of homoeologs along this branch (Fig. 4). This result is the more striking because the inferred mean K_s_ value for the root branch ($\bar{K_{s}^{R}}$) should, in the case of an allopolyploidy, also include the prepolyploidy progenitor divergences. Hence, the $\bar{K_{s}^{R}}$values for these events should be over-estimates, making the $\alpha \cdot t/\bar{K_{s}^{R}}$ ratio an underestimate of the relative homoeolog loss rate along the root branch.

If natural selection were actively favoring the loss of some homoeologous copies immediately after polyploidy, it is possible that the genes involved in those early losses would display a stronger selective constraint than do homoeologous copies lost later in that event’s history due to the possibility of dominant negative interactions or expression-linked dosage conflicts (Drummond *et al.* 2006; De Smet *et al.* 2013; Veitia *et al.* 2013). We hence compared the average selective constraint, measured as the ratio of nonsynonymous to synonymous substitutions (*K_a_*/*K_s_*), of 2 types of fully single-copy genes. The first is the single-copy genes whose homoeolog was lost immediately after the polyploidy event along the root branch; the second is the fully single-copy genes where different extant genomes retain homoeologous copies from alternative subgenomes, a situation that requires that the losses have occurred independently after the first speciation event. For most events, we observe little difference in constraint between these 2 groups, while for the Legume WGD the single-copy genes lost later are actually *more* constrained, the opposite of the prediction for selected losses (Supplementary Fig. 5).

### Extensive reciprocal gene loss between pairs of polyploid taxa

Following Scannell et al. (2006, 2007), we searched for postpolyploidy RGLs. We omitted the vertebrate 2R and nematode triploidy from this analysis due to the fragmented nature of the genomes used. With the exception of 3 closely related yeast species in the *Saccharomyces* genus, every pair of genomes in our remaining eight polyploidies were separated by at least 4 RGLs (this minimal number was seen in the platyfish, tilapia, and medaka clade of the TGD; Fig. 5c), with the number rising to over a thousand for a few of the yeast taxa pairs. These conclusions are also robust to the confidence cutoffs used to infer the RGLs (Supplementary Fig. 6). Our results are in accord with previous work in yeasts and grasses (Scannell *et al.* 2006, 2007; Schnable *et al.* 2012), and there appears to be a relatively direct relationship between the synonymous divergence of a pair of taxa (a proxy for divergence time) and the number of RGLs separating them (Fig. 4, a and b). Such a relationship would be expected if both RGLs and synonymous substitutions were accumulating through neutral evolutionary processes (Fig. 5a). However, the proportionality between synonymous substitutions and RGLs differs between polyploidy events, with the yeast WGD showing more RGLs per unit K_s_ than the other events. When we compared the genes involved in reciprocal losses in zebrafish, *A. thaliana* and bakers’ yeast to other single-copy genes, there were no significant functional differences between these 2 sets, again as one would expect were RGL a neutral process (see *Methods*).

The evolutionary importance of RGLs can be assessed by the biological role of the genes that experienced it. For instance, were only “nonessential” genes to experience RGL, then it might not present significant barriers to hybridization. On the other hand, 2 populations separated by a single RGL for an essential gene would form diploid hybrids whose gametes would lack the gene in question 25% of the time. We can use experimental data on gene essentiality from bakers’ yeast, *A. thaliana* and zebrafish (see *Methods*) to ask whether the proportion of RGLs that include an essential gene differs from the overall proportion of essential single-copy genes. For the At-α and TGD events, the proportion of RGLs where the surviving gene in *A. thaliana* or zebrafish is essential does not differ from the proportion of other single-copy genes that are essential (Supplementary Table 7). Curiously, the RGLs found when comparing bakers’ yeast to some of its nearer relatives are actually *more* likely to be essential than other single-copy genes (Supplementary Table 7). This overrepresentation is likely due to the fact that the duplicate losses that occurred before the first speciation event were actually underrepresented in essential genes (Supplementary Table 8). As a result, RGLs, which must have occurred *after* the first speciation event (see the yeast clade of Fig. 3), would be enriched in essential genes simply because more essential genes survived in duplicate past that first speciation.

The importance of RGL in driving speciation events among polyploid taxa has been questioned on theoretical grounds, as the appearance of RGLs is subject to the same requirement of reproductive isolation as are the appearances of other genetic incompatibilities among populations (Muir and Hahn 2015). This objection has more force for obligately sexual organisms than it does for organisms such as bakers’ yeast, where it is estimated that there are 1,000 mitotic cell divisions for every meiosis and that only about 1% of meioses are out-crosses (Tsai *et al.* 2008). Indeed, Fig. 5 suggests that RGL may occur more frequently in yeasts (and potentially in some plants, which may also reproduce asexually) than in the teleost fishes and particularly the salmonids.

Even if RGL does not drive speciation, it still represents a barrier to diploid hybrids: most of the taxa pairs for which essentiality data are available are separated from each other by at least one RGL for an essential gene, the exceptions being some of the closest relatives of *A. thaliana*, zebrafish and bakers’ yeast studied (Supplementary Table 7). This observation is consistent with studies of the relative frequency of diploid and polyploid hybridizations in flowering plants. In these lineages, it is rare to find successful diploid hybrids involving distantly related parental species (where RGLs could be common). However, allopolyploid hybrids appear to form at roughly the same rate across a much larger range of divergence times (Buggs *et al.* 2009b). A potential explanation for the frequency of recurrent polyploidy is therefore simply that a new allopolyploidy can allow paleopolyploids to again enjoy the benefits of hybridization (such as hybrid vigor and heterosis; Birchler *et al.* 2006; Chen 2010) in the face of their isolation due to RGL.

## Discussion

There are a surprising number of similarities seen in the manner of polyploidy resolution across these independent polyploidies. Biased fractionation and other patterns in the homoeolog losses are similar across many events: RGLs are also present for most pairs of polyploid taxa. The rate of homoeolog loss immediately after polyploidy is very high for many, but not all, events (Fig. 4).

Moreover, the differences in evolutionary patterns we do see are often in keeping with what we know about the history of the events themselves. For instance, the salmonid WGD is marked by continuing pairing of homoeologous chromosomes in meiosis (Allendorf *et al.* 2015). These pairings appear to limit the number of homoeolog losses, and, for this event, loss rates at the phylogeny tips and root are similar (per unit *K_s_*). The grass ρ and yeast events have loss rates that are roughly similar (again per unit *K_s_*) across time, a fact for which we currently do not have an operating hypothesis.

For the events that do show rapid losses along the root branch, which of the 2 hypotheses mentioned, drift or selected losses, seems to better explain our data? The homoeologs lost along the root are not more selectively constrained than other purely single-copy genes known to have been lost later (Supplementary Fig. 5). This fact probably speaks against any very large number of selected losses. The single-copy genes as a whole are also generally somewhat less selectively constrained than are genes with surviving homoeologs (Supplementary Fig. 5). Moreover, there is a clear pattern in most events whereby most of the fully single-copy genes that exist today are predicted to have lost their homoeologous partner along the root branch (Supplementary Fig. 4). The yeast, nematode, and Paramecium events may violate this pattern because the nematode event is an asexual triploidy while the other 2 involve lineages that have significant rates of asexual reproduction. In such cases, restoring proper meiotic pairing is less necessary than in taxa with primarily sexual reproduction. As a result, we expect that asexually reproducing lineages could more easily form viable new species immediately after polyploidy, meaning that the postpolyploid “lag” in speciation might be less evident (Schranz *et al.* 2012). As a preliminary hypothesis, we, therefore, propose that, for most polyploidies in animals and plants, the majority of the purely neutral homoeolog losses occur before extensive species divergence in the polyploid clade. A natural extension to this proposal would be that the postpolyploidy lag represents this earlier period of neutral homoeolog loss, though the question of why speciation events might be rare during such a period is still to be answered. A further implication would be that later losses (including RGLs) would have occurred in homoeologous pairs that were initially preserved to maintain dosage balance. They are then only lost when later mutations, such as expression changes, release this dosage constraint and allow the loss of one of the copies (Birchler *et al.* 2005; Conant *et al.* 2014). The higher selective constraint of genes with surviving homoeologs is arguably also consistent with this hypothesis.

While the best-studied ancient polyploidy is in bakers’ yeast, it is atypical in a number of respects. Biased fractionation is much less evident here (Emery *et al.* 2018), losses are not heavily biased toward the earliest phases of the polyploidy (Fig. 4) and RGL is much more prevalent. As mentioned above, one major source of these differences is likely the relative timing of the postpolyploidy speciations: the yeasts had almost no lag between their polyploidy event and the first observed speciation in our dataset (Supplementary Fig. 4; Schranz *et al.* 2012).

Other questions remain unanswered. The relative formation rates of allo- and autopolyploids are uncertain. While recent polyploids appear to be approximately equally divided between the two (Barker *et al.* 2016), the potential selective advantages of being an allopolyploid, and hence a hybrid (Alix *et al.* 2017; Blanc-Mathieu *et al.* 2017), could result in a strong skew toward allopolyploids among the rare polyploidies that survive to became the ancient events of the kind studied here (Barker *et al.* 2016). The results here are consistent with this hypothesis, but our sample of events is potentially biased by the available genome sequences. Across all of the events, we find that the ubiquity of homoeolog fixation and (except in paramecia) convergent homoeolog losses both speak to a common selective environment acting to maintain certain homoeologs after all of these events. The most obvious candidate for such a selective force is again the dosage balance hypothesis: it argues that highly interacting genes tend to remain in multiple copies postpolyploidy to preserve the stoichiometry of those interactions (Birchler *et al.* 2005; Birchler and Veitia 2012; Tasdighian *et al.* 2017). Whatever the role of RGL in speciation, it is clear that all of these polyploid organisms possess a degree of isolation due to it. The role of RGL in recurrent polyploidy is hence an important topic for future research. Biology has a history of viewing “rules” as being more honored in the breach, but the commonalities in postpolyploidy genome evolution across wide taxonomic distances are both interesting in their own right and for the insight they give on other aspects of biology (Pires and Conant 2016).

## Data availability

All underlying data are available from the POInT browser (wgd.statgen.ncsu.edu; accessed 2022 Apr 28) and from figshare (DOI: https://doi.org/10.6084/m9.figshare.12750992.v4; accessed 2022 Apr 28); the POInT package (v1.55) is available from GitHub (https://github.com/gconant0/POInT; accessed 2022 Apr 28).

Supplemental material is available at *G3* online.

## Supplementary Material

jkac094_Supplementary_Figures_1-6

jkac094_Supplementary_Table_1-8
