# Supplementary material for: Convergent evolution of polyploid genomes from across the eukaryotic tree of life
Source: G3 (Bethesda). 2022 Apr 22;12(6):jkac094. doi: 10.1093/g3journal/jkac094 (PMC9157103; doi:10.1093/g3journal/jkac094)
Supplement: jkac094_Supplementary_Figures_1-6 [file jkac094_supplementary_figures_1-6.pdf]

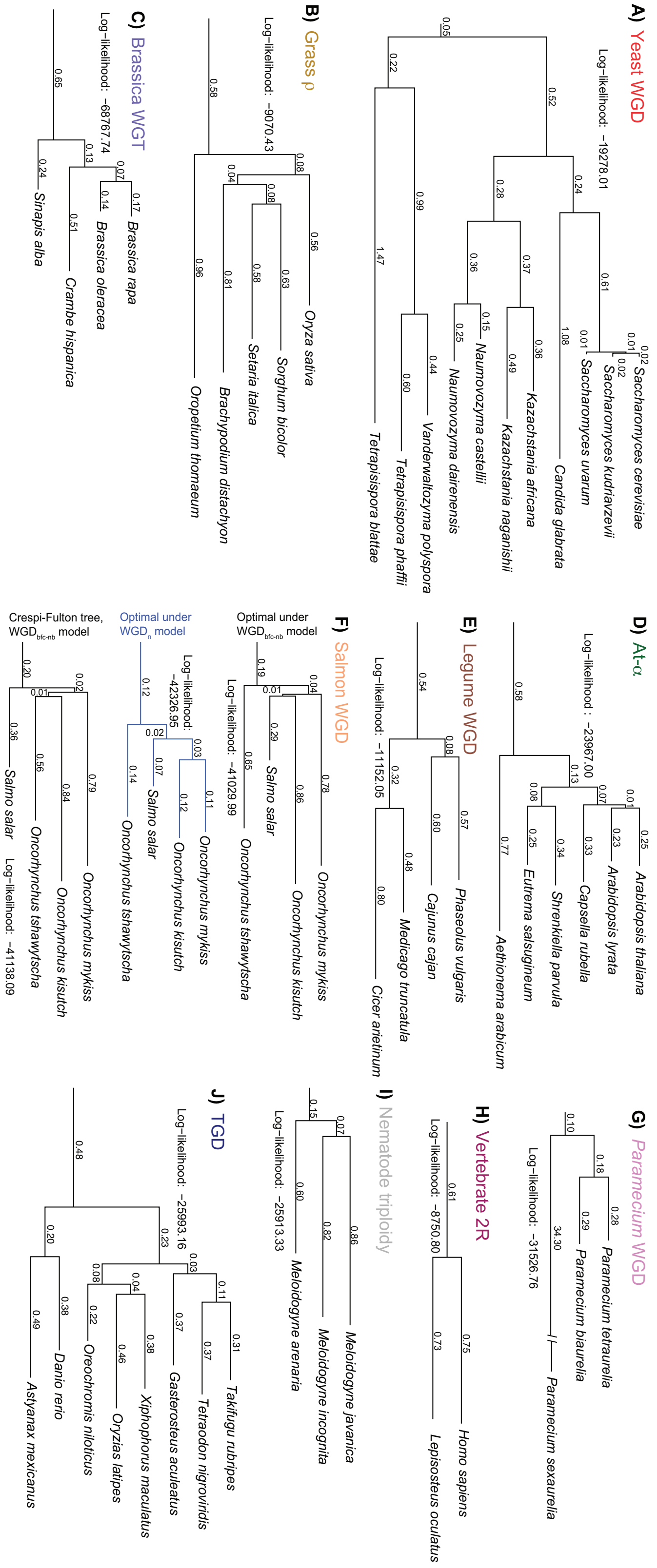

Supplemental Figure 1: Inferred or assumed phylogenetic topologies from POInT for the ten polyploidies, with homeolog loss rates described by branch length. The lengths given above each branch are reported in terms of the base homoeolog loss rate α from Figure 2, multiplied by the estimated time

encompassed by the branch in question (αt): this form is completely analogous to models of DNA sequence evolution where branch lengths are represented in units of μt (HILLIS *et al.* 1996). The likelihood of each tree under the optimal model is given (Supplemental Tables 1&2, with a null model, e.g., Figure 2B, for the vertebrate 2R event and the tree shown in blue for the salmonid WGD). Topologies for the yeast WGD (**A**), At-α (**D**), Brassica WGT (**C**) and TGD (**J**) were taken from the literature (*Methods*), while the topologies for grass ρ (**B**), legume WGD (**E**), *Paramecium* WGD (**G**), and the nematode triploidy (**I**) were inferred using POInT. The vertebrate 2R event (**H**) has a trivial topology. For the salmonid WGD (**F**), three topologies are illustrated. At the top is the optimal tree under the $WGD_{bfc\text{-}nb}$ model. The next tree is the optimal one under the WGD_n model (e.g., a model without biased fractionation, homoeolog fixation or convergent losses). This tree is illustrated with blue branches to indicate that its branch lengths are not drawn to scale (since their interpretation differs due to the different model). Finally, the topology inferred through conventional phylogenetic methods (CRESPI AND FULTON 2004), optimized under the $WGD_{bfc\text{-}nb}$, is also shown. The tree visualizations were made with custom software to accurately represent the length of the root branch (CONANT AND WOLFE 2008).

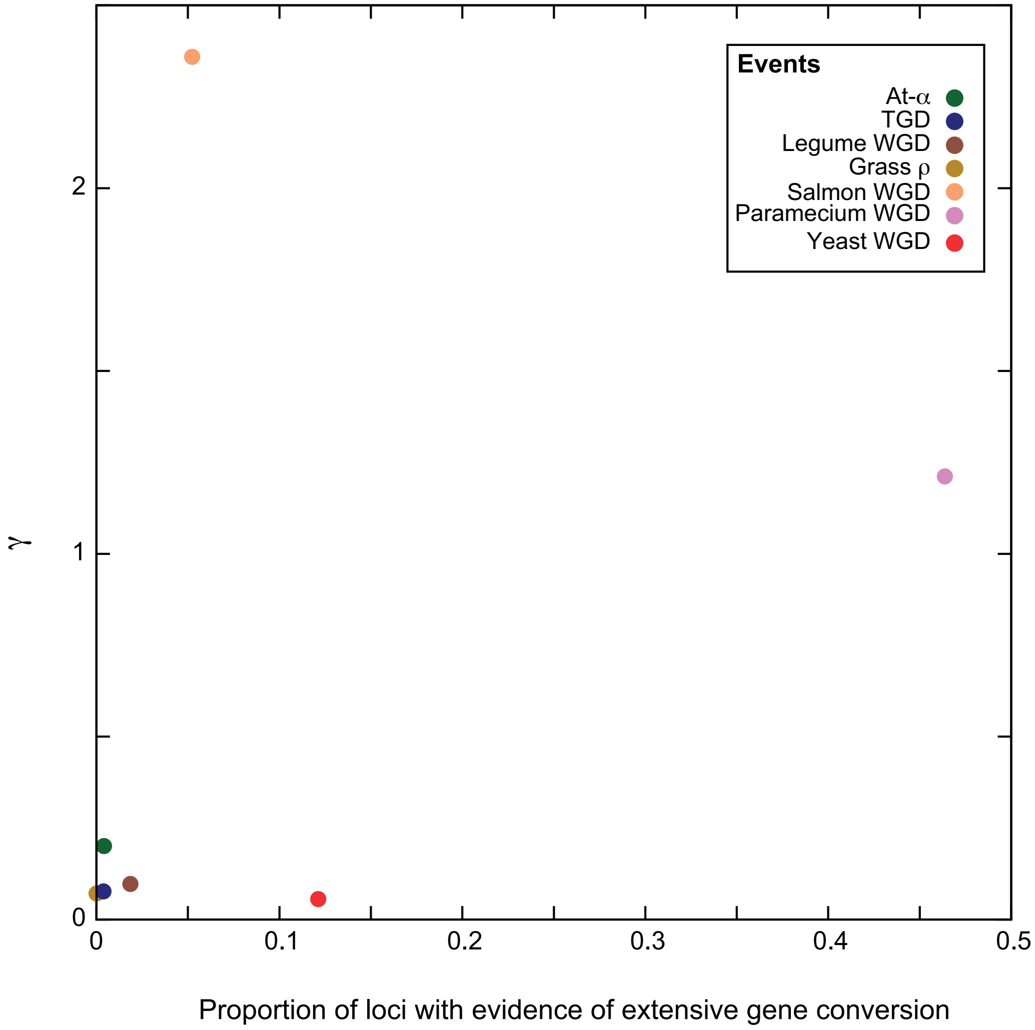

Supplemental Figure 2: For the seven tetraploidies, the *x*-axis gives proportion of surviving fully-retained homoeologous loci where gene trees that pair the homoeologs in each genome with each other (e.g., as expected for very recent gene conversions in all genomes) is favored over mirrored copies of the species tree (*Methods*). On the *y*-axis is POInT's estimate of the rate of duplicate gene fixation relative to duplicate loss for these events (c.f., the purple arrows in Figure 2)

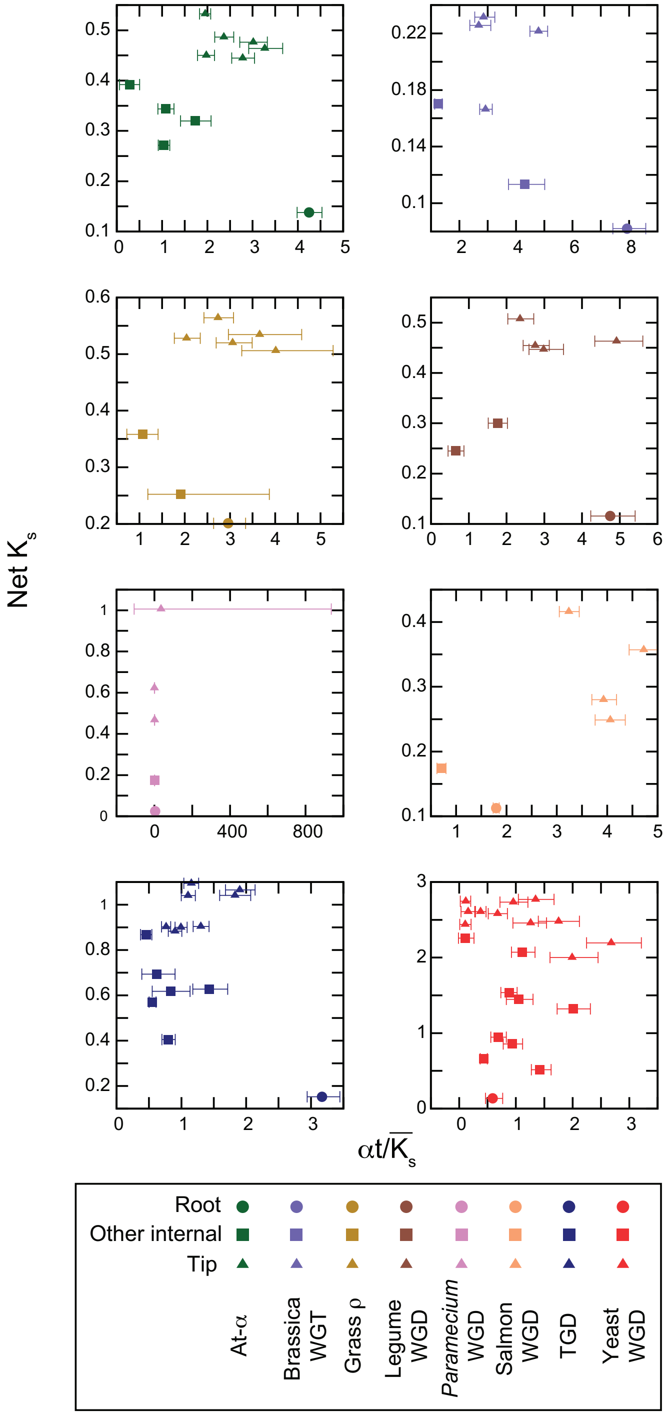

Supplemental Figure 3: The signal of rapid homoeolog loss observed on the root branch relative to synonymous divergence is also seen with the full $WGD_{bfc\text{-}nb}$ model (c.f., to Figure 3). The inclusion of fixation of duplicate in this model can give rise to elevated estimates of αt along tip branches, as is seen for one branch each in the Legume and Paramecium WGD events. Otherwise, the signal of rapid loss along the root is seen as previously. The *x*-axis gives the ratio of rate of homoeolog loss to synonymous divergence for a branch (*Methods*) and the *y*-axis is the net synonymous divergence to the end of the branch in question. The root branch is indicated with a circle, other internal branches with squares and tip branches with triangles. 95% confidence intervals for our estimates of $\alpha t/K_s$ were computed as described in the *Methods*.

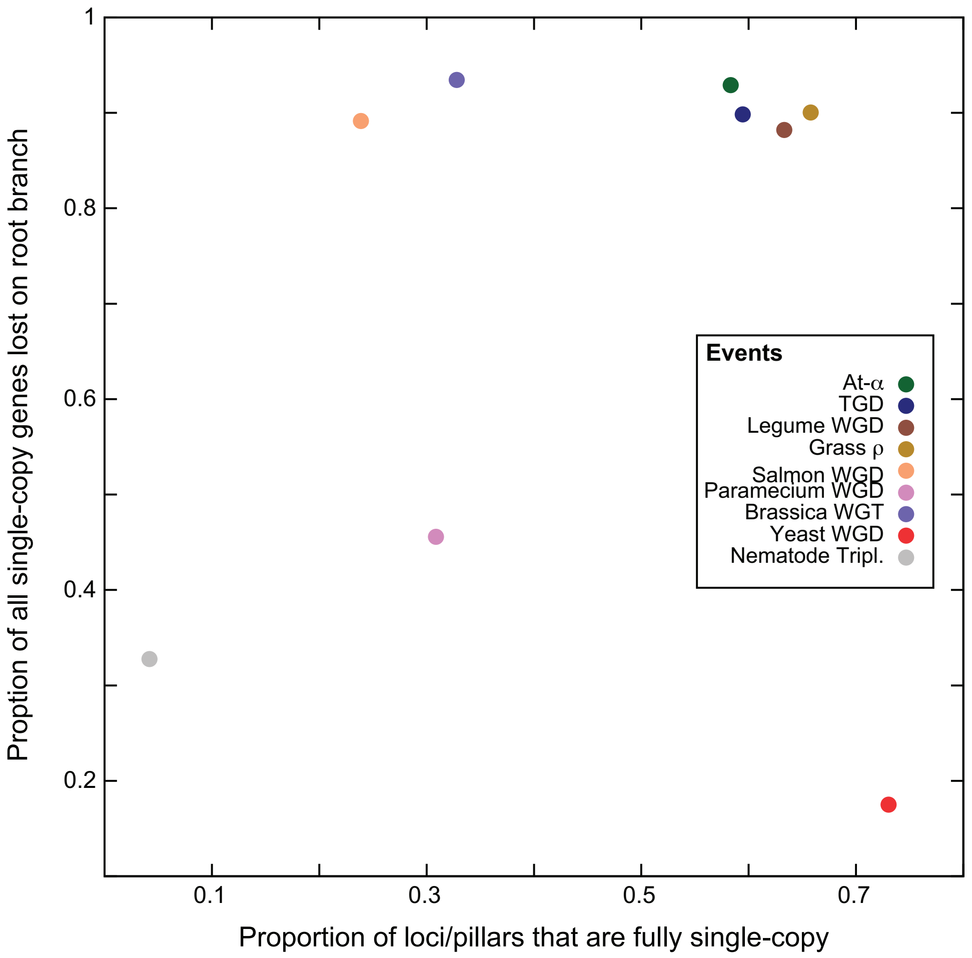

Supplemental Figure 4: Comparisons of the proportion of the homoeologous loci (e.g., pillars) that are single-copy in all surveyed genomes (*x*-axis) to the proportion of those fully single-copy loci that POInT predicts were returned to single-copy along the shared root branch (in other words, prior to the first speciation event among the surveyed genomes; *y*-axis). This second figure is computed as the sum of conditional probabilities of a loss event along the common root branch across all of the pillars in the dataset. Hence, if two pillars are each predicted to have had a 50% chance of a loss on the root branch, we would infer one loss between the two of them (0.5 + 0.5 =1.0).

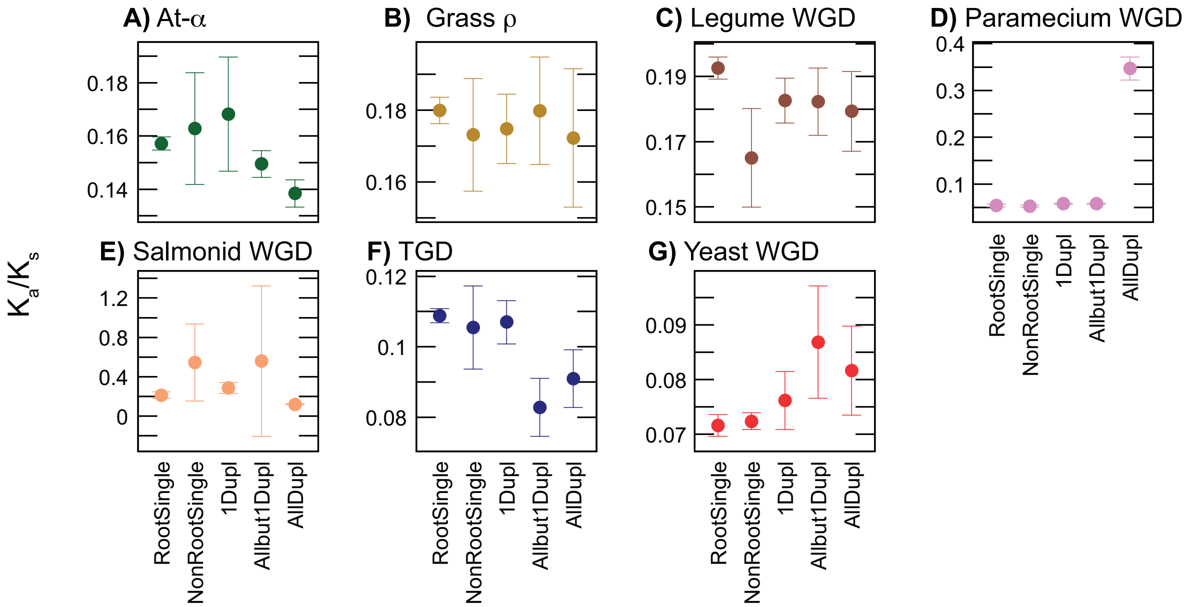

Supplemental Figure 5: Selective constraints of polyploid loci with different degrees of homoeolog preservation. For each of the seven tetraploidies analyzed, we compare the inferred average selective constraint of five classes of homoeologous loci/pillars: 1) pillars where POInT predicts that the homoeologous copy was lost on the common root branch (RootSingle) and hence all genomes have single-copy orthologs at the locus in questions, 2) Pillars where all genomes have only a single copy of the gene but where POInT predicts the loss was unlikely (P<0.1) to have occurred on the root branch (NonRootSingle), 3) Pillars with one taxa having a duplicate copy present and the remaining genomes having only a single gene present (1Dupl), 4) Pillars where all genomes save one retain the duplicate (Allbut1Dupl) and 5) Pillars where all genomes retain the duplicate copy (AllDupl). For #1 (RootSingle), we used a cutoff of P>0.9 confidence in the root loss for all events except the Paramecium WGD (P>0.7) and the yeast WGD (P>0.4). The lower cutoff for the Paramecium event was necessitated because we only analyzed three genomes with the WGD; for the yeast WGD the very rapid post-WGD split among the taxa (see Supplemental Figure 4) meant that our confidence in when genes were lost was much lower than for the other events.

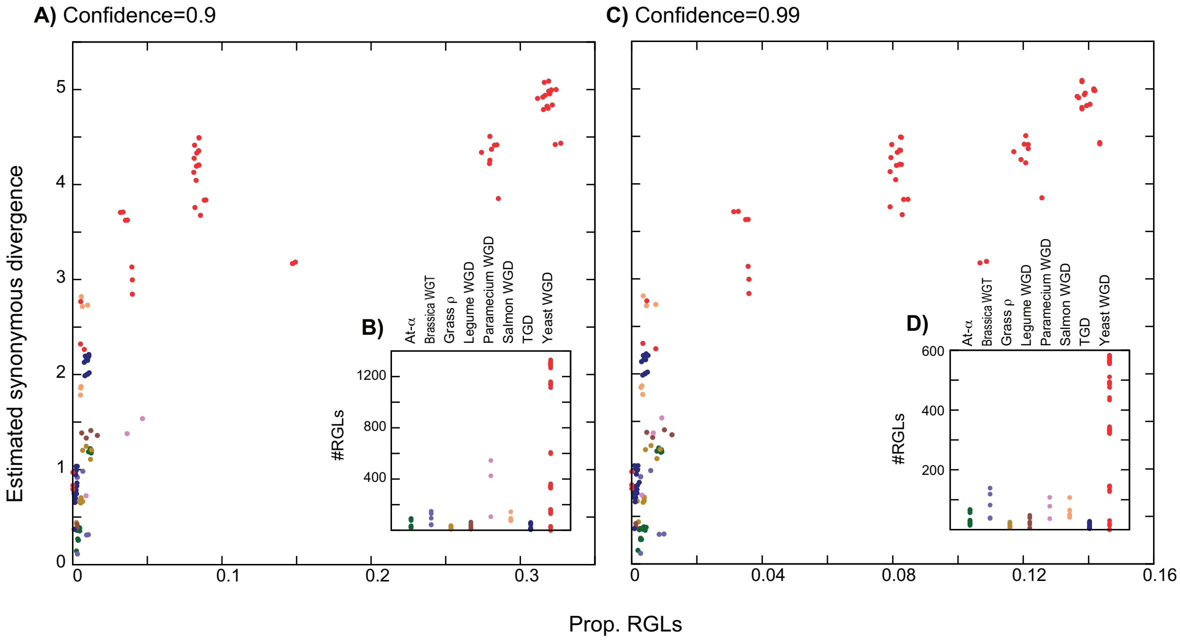

Supplemental Figure 6: Inference of RGLs between polyploid taxa is robust to the choice of POInT's orthology confidence cutoff. Panels **A** and **C** are created as for Figure 4A, except that the orthology confidence used in **A** was 90% and was 99% in **C.** Panels **B** and **D** are similarly constructed relative to Figure 4B.

References:

Conant, G. C., and K. H. Wolfe, 2008 Probabilistic cross-species inference of orthologous genomic regions created by whole-genome duplication in yeast. Genetics 179**:** 1681-1692.

Crespi, B. J., and M. J. Fulton, 2004 Molecular systematics of Salmonidae: combined nuclear data yields a robust phylogeny. Molecular phylogenetics and evolution 31**:** 658-679.

Hillis, D. M., C. Moritz and B. K. Mable, 1996 *Molecular Systematics: Second Edition*. Sinauer Associates, Sunderland, MA.
