# Supplementary material for: Convergent evolution of polyploid genomes from across the eukaryotic tree of life
Source: G3 (Bethesda). 2022 Apr 22;12(6):jkac094. doi: 10.1093/g3journal/jkac094 (PMC9157103; doi:10.1093/g3journal/jkac094)
Supplement: jkac094_Supplementary_Table_1-8 [file jkac094_supplementary_table_1-8.docx]

**Supplemental Table 1: Polyploid genomes analyzed**

| **Event** | **Species** | **Genome reference** | **Genome source/Accession** |
| --- | --- | --- | --- |
| At-α | *Arabidopsis thaliana* | ^1^ | CoGe^2^: 20342 |
|  | *Arabidopsis lyrata* | ^3^ | CoGe^2^: 3068 |
|  | *Capsella rubella* | ^4^ | CoGe^2^: 16754 |
|  | *Shrenkiella parvula* | ^5^ | CoGe^2^: 12384 |
|  | *Eutrema salsugineum* | ^6^ | CoGe^2^: 19492 |
|  | *Aethionema arabicum* | ^7^ | CoGe^2^: 23428 |
| Brassica WGT | *Brassica rapa* | ^8^ | CoGe^2^: 24668 |
|  | *Brassica oleracea* | ^9^ | CoGe^2^: 26018 |
|  | *Sinapis alba* | Unpublished | CoGe^2^: 33284 |
|  | *Crambe hispanica* | ^10^ | CoGe^2^: 58014 |
| Grass ρ | *Brachypodium distachyon* | ^11^ | CoGe^2^: 25040 |
|  | *Oropetium thomaeum* | ^12^ | CoGe^2^: 25799 |
|  | *Setaria italica* | ^13^ | CoGe^2^: 28806 |
|  | *Sorghum bicolor* | ^14^ | CoGe^2^:31607 |
|  | *Oryza sativa* | ^15^ | CoGe^2^: 16888 |
| Legume WGD | *Cajunus cajan* | ^16^ | CoGe^2^: 12470 |
|  | *Cicer arietinum* | ^17^ | CoGe^2^: 32935 |
|  | *Medicago truncatula* | ^18^ | CoGe^2^: 28969 |
|  | *Phaseolus vulgaris* | Unpublished | CoGe^2^: 37301 |
| TGD | *Astyanax mexicanus* | ^19^ | Ensembl release 84^20^ |
|  | *Danio rerio* | ^21^ |  |
|  | *Takifugu rubripes* | ^22^ |  |
|  | *Oryzias latipes* | ^23^ |  |
|  | *Xiphophorus maculatus* | ^24^ |  |
|  | *Gasterosteus aculeatus* | ^25^ |  |
|  | *Tetraodon nigroviridis* | ^26^ |  |
|  | *Oreochromis niloticus* | ^27^ |  |
| Salmon WGD | *Salmo salar* | ^28^ | NCBI assembly: GCF_000233375.1 |
|  | *Oncorhynchus mykiss* | ^29^ | NCBI assembly: GCF_002163495.1 |
|  | *Oncorhynchus kisutch* | Unpublished | NCBI assembly: GCF_002021735.1F_002021735.1 |
|  | *Oncorhynchus tshawytscha* | ^30^ | NCBI assembly: GCF_002872995.1 |
| *Paramecium* WGQ/WGD | *Paramecium tetraurelia* | ^31^ | ParameciumDB ^32^ |
|  | *Paramecium biaurelia* | Unpublished | ParameciumDB ^32^, v. 1-4 |
|  | *Paramecium sexaurelia* | Unpublished | ParameciumDB ^32^, v AZ8-4 |
| Nematode triploidy | *Meloidogyne arenaria* | ^33^ | https://meloidogyne.inrae.fr |
|  | *Meloidogyne incognita* | ^33^ |  |
|  | *Meloidogyne javanica* | ^33^ |  |
| Vertebrate 2R | *Homo sapiens* | ^34,35^ | Ensembl release 98^20^ |
|  | *Lepisosteus oculatus* | ^36^ |  |
| Yeast WGD | *Saccharomyces cerevisiae* | ^37^ | YGOB ^38,39^ |
|  | *Saccharomyces kudriavzevii* | ^40^ |  |
|  | *Saccharomyces uvarum* | ^40^ |  |
|  | *Candida glabrata* | ^41^ |  |
|  | *Naumovozyma castellii* | ^42^ |  |
|  | *Naumovozyma dairenensis* | ^42^ |  |
|  | *Kazachstania africana* | ^42^ |  |
|  | *Kazachstania naganishii* | ^42^ |  |
|  | *Vanderwaltozyma polyspora* | ^43^ |  |
|  | *Tetrapisispora phaffii* | ^42^ |  |
|  | *Tetrapisispora blattae* | ^42^ |  |

**Supplemental Table 2: Homology inference**

| **Event** | **BLAST E-value≤^a^** | **%ID^a^** | **% Length^a^** | **Outgroup Genome^b^** | **Pillars/Synteny breaks^c^** |
| --- | --- | --- | --- | --- | --- |
| Yeast WGD | NA^d^ | NA^d^ | NA^d^ | NA^d^ | 4065/4346 |
| At-α | NA^e^ | 70% | 80% | *Cleome violacea^44^* | 7243/3586^e^ |
| TGD | 10^-8^ | 60% | 65% | *Lepisosteus oculatus^36^* | 5589/14,370 |
| Brassica WGT | 10^-10^ | 70% | 80% | *Arabdiposis thaliana^1^* | 14,050/5237 |
| Legume WGD | 10^-10^ | 70% | 80% | *Cucumis melo^45^* | 3886/3521 |
| Grass ρ | 10^-10^ | 70% | 80% | *Ananas comosus^46^* | 2855/2842^f^ |
| *Paramecium* WGQ | 10^-7^ | 70% | 80% | *Paramecium caudatum^47^* | 11683/1584 |
| Nematode triploidy | 10^-9^ | 70% | 80% | *Meloidogyne hapla^48^* | 5316/12,724 |
| Salmon WGD | 10^-8^ | 70% | 80% | *Esox lucius^49^* | 14,489/5239 |
| Vertebrate 2R | 10^-5^ | 40% | 80% | *Ciona intestinalis^50^* | 2570/2603 |

^a:^ GenomeHistory^51^ with the indicated BLASTP^52^ E-value thresholds and pairwise amino acid percent identity cutoff was used to identify pairs of homologous genes between the non-polyploid relative (Outgroup Genome, right) and each polyploid genome. Only alignments spanning the indicated percentage of the shorter sequence were accepted.

^b:^ The indicated genomes which lack the respective polyploidies served as the basis of a homology search to each of the polyploid genomes.

^c:^The number of polyploid loci or “pillars” is given first (c.f., Figure 2A). The number of synteny breaks is the number of positions where two genes that are neighbors in a pillar are not neighbors in a particular modern genome.

^d:^ Yeast DCS inferences were taken from YGOB and the inferences of Gordon et al., ^38,53^

^e:^ For At-α we used a proprietary search algorithm bases on the SeqAn package ^54^ as described in Emery et al., ^44^ However, the ancestral order for these six genomes was reoptimized for this work, reducing the number of breakpoints from the 5468 reported in Emery et al., to the 3586 reported here.

^f:^ The analysis of the grass ρ event presented here differs from our prior publication*^44^* because it includes the rice genome in addition to the four presented there.

**Supplemental Table 3: Nested models of post-tetraploidy gene loss**

| **Event** | **lnL WGD_n_** | **lnL WGD_f_** | ***P ^a^*** | **lnL WGD_b_** | ***P ^a^*** | **lnL WGD_c_** | ***P ^b^*** | **lnL WGD_bfc_ ^c^** | ***P ^d^*** | **lnL WGD_bfc-nb_ ^c^** | ***P ^e^*** |
| --- | --- | --- | --- | --- | --- | --- | --- | --- | --- | --- | --- |
| Yeast WGD | -19870.9 | -19374.1 | *<10^-10^* | -19870.9 | *>0.5* | -19062.8 | *<10^-10^* | NA | *<10^-10^* | NA | NA |
| At-α | -24562.4 | -24417.6 | *<10^-10^* | -24462.4 | *<10^-10^* | -24142.8 | *<10^-10^* | -23965.5 | *<10^-10^* | -23967.0 | 0.23 |
| TGD | -26379.3 | -26291.0 | *<10^-10^* | -26343.2 | *<10^-10^* | -26061.4 | *<10^-10^* | -25992.4 | *<10^-10^* | -25993.2 | 0.47 |
| Legume WGD | -11279.0 | -11252.5 | *<10^-10^* | -11264.2 | *6x10^-8^* | -11195.4 | *<10^-10^* | -11151.1 | *<10^-10^* | -11152.1 | 0.38 |
| Grass ρ | -9299.9 | -9260.3 | *<10^-10^* | -9285.4 | *8x10^-8^* | -9115.0 | *<10^-10^* | -9070.1 | *<10^-10^* | -9070.4 | 0.71 |
| *Paramecium* WGD | -31912.2 | -31570.1 | *<10^-10^* | -31866.5 | *<10^-10^* | -31570.1 | *<10^-10^* | -31526.8^f^ | *<10^-10^* | NA | NA |
| Salmon WGD | -42571.1 | -41578.1 | *<10^-10^* | -42485.0 | *<10^-10^* | -41607.2 | *<10^-10^* | -41030.0 | *<10^-10^* | -41030.0 | 0.97 |

*^a:^* Likelihood ratio test between the WGD_n_ (γ=0, δ=0, ε=1) and WGD­_f_ (γ≥0, δ=0, ε=1) or WGD_b_ (γ=0, δ=0, ε≤1) models, d.f.=1*.* See Figure 2.

*^b:^* Likelihood ratio test between the WGD_n_ and WGD­_c_ (γ=0, δ≥0, ε=1) models, d.f.=2*.*

*^c:^*The WGD_bfc_ and the WGD_bfc-nb_ models differ in that the latter does not include a subgenome bias in the formation of the “converging duplicates” (states C_1_ and C_2_ in Figure 2) and the loss rate from C_1_ and C_2_ are assumed to be equal to that from U.

*^d:^* Likelihood ratio test between the WGD_n_ and WGD­_bfc_ models, d.f.=5*.*

*^e:^* Likelihood ratio test between the WGD_bfc-nb_ and WGD­_bfc_ models, d.f.=2*.*

*^f:^* For the *Paramecium* WGD, the fit of the pillar data to the WGD_bfc_ model was not significantly better than when using a model with only fixation and bias included (*P>0.5*; WGD_bf_): the maximum ln-likelihood of the latter model was also -31526.8.

**Supplemental Table 4: Nested models of post-hexaploidy gene loss**

| **Event** | **lnL WGT_n_** | **lnL WGT_G2_** | ***P ^a^*** | **lnL WGT_G3_** | ***P ^b^*** | **lnL WGT_G3+R_** | ***P ^c^*** |
| --- | --- | --- | --- | --- | --- | --- | --- |
| Brassica WGT | -69906.4 | -68940.8 | *<10^-10^* | -68855.8 | *<10^-10^* | -68767.7 | *<10^-10^* |
| Nematode triploidy | -27312.2 | -26714.4 | *<10^-10^* | -26069.7 | *<10^-10^* | -25913.3 | *<10^-10^* |

*^a:^* Likelihood ratio test between the WGT_n_ and WGT­_G2_ models, d.f.=2*.* WGT_n_ assumes equal loss rates from all subgenomes (*f_1,3_=f_2,3_*=1.0, σ_1_=σ_2_= σ _3_). WGT­_G2_ assumes equal loss rates from subgenomes 2 and 3 (*f_1,3_*=1.0, σ_2_= σ _3_). See Figure 2 for details.

*^b:^* Likelihood ratio test between the WGT_n_ and WGT­_G3_ models, d.f.=4*.*

*^c:^* Likelihood ratio test between the WGT_G3_ and WGT­_G3-R_ models, d.f.=5 (see Figure 2).

**Supplemental Table 5: Biased fractionation and convergent gene losses**

| **Event** | **lnL WGD_f_^a^** | **lnL WGD_bf_^a^** | **ε** | ***P ^b^*** | **lnL WGD_bf-root_^c^** | **ε_other_/**  **ε_root_** | ***P ^d^*** | **lnL WGD_bfc-nb_ ^e^** | **ε** |
| --- | --- | --- | --- | --- | --- | --- | --- | --- | --- |
| At-α | -24417.6 | -24316.1 | 0.68 | <10^-15^ | -24314.1 | 0.70/0.64 | 0.04 | -23967.0 | 0.56 |
| TGD | -26291.0 | -26253.7 | 0.75 | <10^-15^ | -26244.5 | 0.67/0.87 | <10^‑4^ | -25993.2 | 0.71 |
| Legume WGD | -11252.5 | -11237.1 | 0.76 | <10^-8^ | -11235.8 | 0.70/0.81 | 0.1 | -11152.1 | 0.70 |
| Grass ρ | -9258.7 | -9242.6 | 0.77 | <10^-8^ | -9240.6 | 0.70/0.83 | 0.04 | -9070.4 | 0.75 |
| *Paramecium* WGD | -31570.1 | -31526.8 | 0.73 | <10^-15^ | -31477.03 | 0.88/0.35 | <10^-15^ | NA^e^ | NA^e^ |
| Salmon WGD | -41535.7 | -41451.1 | 0.72 | <10^-15^ | -41437.8 | 0.80/0.54 | <10^-6^ | -410230.0 | 0.56 |

*^a:^* The WGD_bf_ model adds a biased fractionation parameter ε to the WGD_f_ model (with duplication fixation only)

*^b:^* Likelihood ratio test between the WGD_f_ (γ≥0, δ=0, ε=1) and WGD­_bf_ (γ≥0, δ=0, ε≤1), d.f.=1*.*

*^c:^* The WGD_bf_ and the WGD_bf-root_ models differ in that the latter allows a different value of ε for the root branch (ε_root_) relative to all other branches (ε_other_).

*^d:^* Likelihood ratio test between the WGD_bf_ and WGD­_bf-root_ models, d.f.=1*.*

^e:^ No significant pattern of convergent losses was observed for the *Paramecium* WGD (Supplemental Table 1).

**Supplemental Table 6: Little evidence of bimodal synonymous divergence on root branches**

| **Event** | **Dataset^a^** | **Preferred Model^b^** | **Mean K_s_^l^/Mean K_s_^h c^** | **Prop. K_s_^l^/ Prop. K_s_^h d^** |
| --- | --- | --- | --- | --- |
| At-α | 0.95 | Unimodal | - | - |
|  | 0.95, fully duplicated | Unimodal | - | - |
| Brassica WGT | 0.95 ^e^ | Bimodal | 0.008/0.071 | 0.041/0.959 |
|  | 0.99 ^e^ | Bimodal | 0.006/0.068 | 0.023/0.977 |
| Grass ρ | 0.95 | Bimodal | 0.142/0.425 | 0.833/0.166 |
|  | 0.95, fully duplicated | Unimodal | - | - |
| Legume WGD | 0.95 | Bimodal | 0.081/0.381 | 0.921/0.079 |
|  | 0.95, fully duplicated | Bimodal | 0.075/0.328 | 0.933/0.067 |
| Paramecium WGD | 0.95 | Unimodal | - | - |
|  | 0.95, fully duplicated | Bimodal | 0.016/0.093 | 0.953/0.047 |
| Salmon WGD | 0.95 | Unimodal | - | - |
|  | 0.95, fully duplicated | Unimodal | - | - |
| TGD | 0.95 | Unimodal | - | - |
|  | 0.95, fully duplicated | Bimodal | 0.032/0.165 | 0.147/0.852 |
| Yeast WGD | 0.95 | Unimodal | - | - |
|  | 0.95, fully duplicated | Unimodal | - | - |

*^a:^* We tested for evidence of a bimodal distribution of root K_s_ values (see *Methods*) using both a set of nearly fully-duplicated loci at 95% orthology confidence (0.95) and for a reduced set consisting of only fully duplicated loci (0.95, fully duplicated). See note **e** for the Brassica triplication.

*^b:^* Indicates whether the Bayesian Information Criterion (BIC) preferred a model with a single normal distribution of K_s_ values (Unimodal) or a model with two such normal distributions (Bimodal).

*^c:^* In cases where a bimodal distribution was inferred, the estimated smaller and larger mean K_s_ values are given (K_s_^l^ and K_s_^h^, respectively).

*^d:^* In cases where a bimodal distribution was inferred, the estimated proportion of loci belonging to the smaller and larger K_s_ distributions are given.

*^e:^* For the Brassica WGT, only fully triplicated loci were considered. Hence, we give the results of the BIC analysis for orthology confidence cutoffs of 95% and 99% (0.95 and 0.99, respectively).

**Supplemental Table 7: RGL and gene essentiality**

| **Event** | **Species with essentiality data *^a^*** | **Compared species *^b^*** | **RGL *^c^*** | | | **All other single-copy *^d^*** | | | ***P ^e^*** |
| --- | --- | --- | --- | --- | --- | --- | --- | --- | --- |
|  |  |  | **# essential** | **# not**  **essential** | **%**  **essential** | **# essential** | **# not**  **essential** | **%**  **essential** |  |
| Yeast WGD | *S. cerevisiae*^f^ | *C. glabrata* | 70 | 156 | 0.45 | 906 | 3396 | 0.27 | **3x10^‑6^** |
|  |  | *N. castellii* | 143 | 331 | 0.43 | 833 | 3221 | 0.26 | **1x10^‑10^** |
|  |  | *N. dairenensis* | 142 | 337 | 0.42 | 834 | 3215 | 0.26 | **2x10^-9^** |
|  |  | *K. africana* | 149 | 343 | 0.43 | 827 | 3209 | 0.26 | **3x10^-11^** |
|  |  | *K. naganishii* | 148 | 343 | 0.43 | 828 | 3209 | 0.26 | **6x10^-11^** |
|  |  | *V. polyspora* | 260 | 969 | 0.27 | 716 | 2583 | 0.28 | 0.61 |
|  |  | *T. phaffii* | 259 | 971 | 0.27 | 717 | 2581 | 0.28 | 0.53 |
|  |  | *T. blattae* | 229 | 846 | 0.27 | 747 | 2706 | 0.28 | 0.79 |
| At-α | *A. thaliana* | *A. lyrata* | 1 | 18 | 0.06 | 190 | 5149 | 0.04 | 0.49 |
|  |  | *C. rubella* | 0 | 24 | 0 | 191 | 5143 | 0.04 | 1 |
|  |  | *S. parvula* | 1 | 21 | 0.05 | 190 | 5146 | 0.04 | 0.55 |
|  |  | *E. salsugineum* | 0 | 26 | 0 | 191 | 5141 | 0.04 | 0.62 |
|  |  | *A. arabicum* | 3 | 87 | 0.03 | 188 | 5080 | 0.04 | 1 |
| TGD | *D. rerio* | *A. mexicanus* | 0 | 11 | 0 | 146 | 4318 | 0.03 | 1 |
|  |  | *G. aculeatus* | 2 | 55 | 0.04 | 144 | 4274 | 0.03 | 0.71 |
|  |  | *O. niloticus* | 2 | 48 | 0.04 | 144 | 4281 | 0.03 | 0.68 |
|  |  | *O. latipes* | 2 | 50 | 0.04 | 144 | 4279 | 0.03 | 0.69 |
|  |  | *T. nigroviridis* | 2 | 50 | 0.04 | 144 | 4279 | 0.03 | 0.69 |
|  |  | *T. rubripes* | 1 | 46 | 0.02 | 145 | 4283 | 0.03 | 1 |
|  |  | *X. maculatus* | 2 | 47 | 0.04 | 144 | 4282 | 0.03 | 0.67 |

*^a:^* Polyploid species for which essentiality data are available (see *Methods).*

*^b:^* Polyploid species where RGLs were identified relative to the species with essentiality data.

*^c:^* The number of reciprocally-lost single copy genes (RGLs) that are essential, that are not essential (in other words all of the other RGL loci) and the proportion of the RGL loci that are essential (the number of essential RGLs over the total number of RGLs), respectively. The data shown are for the set of inferred RGLs at 95% confidence (see *Methods*).

*^d:^* As for **c,** except computed for all other single-copy loci in the genome for which essentiality data are available.

*^e:^* *P-*values resulting from the test of the null hypothesis of equal proportions of essential genes in the RGL and non-RGL sets (Fisher’s exact test, *Methods*).

*^f:^*Species *S. kudriavzevii* and *S. uvarum* showed no RGLs with *S. cerevisiae* and are omitted from this table.

**Supplemental Table 8: Essentiality and early gene losses**

| **Event** | **Branch^a^** | **Confid.^b^** | **Branch: *#*Essen/Total^c^** | **Prop.^c^** | **Other: *#*Essen/Total^d^** | **Prop.^d^** | ***P ^e^*** |
| --- | --- | --- | --- | --- | --- | --- | --- |
| Yeast WGD | EightSpp Common^f^ | 0.9^g^ | 328/1322 | 0.248 | 648/2230 | 0.291 | 0.006 |
|  | Root | 0.4^g^ | 228/927 | 0.246 | 748/2625 | 0.285 | 0.023 |
| At-α | Root | 0.95 | 81/2308 | 0.035 | 110/2859 | 0.038 | 0.55 |
| TGD | Root | 0.95 | 37/1159 | 0.032 | 109/3170 | 0.034 | 0.78 |

*^a:^* Branch along which the set of genes returned to single-copy was inferred: “Root” refers to the shared branch joining all of the taxa descending from the polyploidy event in question*.*

*^b:^* Probability cutoff for the inference of the timing of gene loss: hence 0.9 implies 90% certainty that the gene in question was returned to single copy along the branch in question.

*^c:^*For the branch in question the number and proportion of those genes that are known to be essential (see Methods) as well as the total number of single-copy genes that returned to single-copy along that branch. The following column gives the resulting proportion of essential genes.

*^d:^* The number of essential genes out of the full set of single-copy genes *not* returned to single copy on the branch in question, as well as that total number of genes returned to single copy on other branches. The following column gives the resulting proportion of essential genes.

*^e:^* *P*-value for the hypothesis test of equal proportions of essential genes in the two groups, Fisher’s exact test*.*

*^f:^* This branch joins *S. cerevisiae* and its seven closest relatives and is marked with a “*” in Figure 1A.

*^g:^*POInT’s ability to pinpoint the timing of early gene losses for the yeast WGD is quite limited, meaning that relaxed confidences were required for these estimates.

**References:**

1. The Arabidopsis Genome Initiative. Analysis of the genome sequence of the flowering plant *Arabidopsis thaliana*. *Nature* **408**, 796-815 (2000).

2. Lyons, E. & Freeling, M. How to usefully compare homologous plant genes and chromosomes as DNA sequences. *The Plant Journal* **53**, 661-673 (2008).

3. Hu, T.T. *et al.* The Arabidopsis lyrata genome sequence and the basis of rapid genome size change. *Nat Genet* **43**, 476-81 (2011).

4. Slotte, T. *et al.* The Capsella rubella genome and the genomic consequences of rapid mating system evolution. *Nat Genet* **45**, 831-5 (2013).

5. Dassanayake, M. *et al.* The genome of the extremophile crucifer Thellungiella parvula. *Nat Genet* **43**, 913-8 (2011).

6. Yang, R. *et al.* The Reference Genome of the Halophytic Plant Eutrema salsugineum. *Front Plant Sci* **4**, 46 (2013).

7. Haudry, A. *et al.* An atlas of over 90,000 conserved noncoding sequences provides insight into crucifer regulatory regions. *Nature Genetics* **45**, 891-898 (2013).

8. Wang, X. *et al.* The genome of the mesopolyploid crop species Brassica rapa. *Nature Genetics* **43**, 1035-9 (2011).

9. Liu, S. *et al.* The Brassica oleracea genome reveals the asymmetrical evolution of polyploid genomes. *Nat Commun* **5**, 3930 (2014).

10. Hao, Y. *et al.* The contributions of the allopolyploid parents of the mesopolyploid Brassiceae are evolutionarily distinct but functionally compatible. *Genome Research* **31**, 799-810 (2021).

11. Initiative, I.B. Genome sequencing and analysis of the model grass Brachypodium distachyon. *Nature* **463**, 763 (2010).

12. VanBuren, R. *et al.* Single-molecule sequencing of the desiccation-tolerant grass Oropetium thomaeum. *Nature* **527**, 508 (2015).

13. Zhang, G. *et al.* Genome sequence of foxtail millet (Setaria italica) provides insights into grass evolution and biofuel potential. *Nature biotechnology* **30**, 549 (2012).

14. Paterson, A.H. *et al.* The Sorghum bicolor genome and the diversification of grasses. *Nature* **457**, 551 (2009).

15. International, R.G.S.P. The map-based sequence of the rice genome. *Nature* **436**, 793 (2005).

16. Varshney, R.K. *et al.* Draft genome sequence of pigeonpea (Cajanus cajan), an orphan legume crop of resource-poor farmers. *Nature biotechnology* **30**, 83 (2012).

17. Varshney, R.K. *et al.* Draft genome sequence of chickpea (Cicer arietinum) provides a resource for trait improvement. *Nature biotechnology* **31**, 240 (2013).

18. Young, N.D. *et al.* The Medicago genome provides insight into the evolution of rhizobial symbioses. *Nature* **480**, 520-524 (2011).

19. McGaugh, S.E. *et al.* The cavefish genome reveals candidate genes for eye loss. *Nature Communications* **5**, 5307 (2014).

20. Aken, B.L. *et al.* Ensembl 2017. *Nucleic Acids Research* **45**, D635-D642 (2017).

21. Howe, K. *et al.* The zebrafish reference genome sequence and its relationship to the human genome. *Nature* **496**, 498-503 (2013).

22. Aparicio, S. *et al.* Whole-genome shotgun assembly and analysis of the genome of Fugu rubripes. *Science* **297**, 1301-10 (2002).

23. Kasahara, M. *et al.* The medaka draft genome and insights into vertebrate genome evolution. *Nature* **447**, 714-9 (2007).

24. Schartl, M. *et al.* The genome of the platyfish, Xiphophorus maculatus, provides insights into evolutionary adaptation and several complex traits. *Nature Genetics* **45**, 567-72 (2013).

25. Jones, F.C. *et al.* The genomic basis of adaptive evolution in threespine sticklebacks. *Nature* **484**, 55-61 (2012).

26. Jaillon, O. *et al.* Genome duplication in the teleost fish Tetraodon nigroviridis reveals the early vertebrate proto-karyotype. *Nature* **431**, 946-57 (2004).

27. Brawand, D. *et al.* The genomic substrate for adaptive radiation in African cichlid fish. *Nature* **513**, 375-381 (2014).

28. Lien, S. *et al.* The Atlantic salmon genome provides insights into rediploidization. *Nature* **533**, 200-205 (2016).

29. Berthelot, C. *et al.* The rainbow trout genome provides novel insights into evolution after whole-genome duplication in vertebrates. *Nature communications* **5**, 1-10 (2014).

30. Christensen, K.A. *et al.* Chinook salmon (Oncorhynchus tshawytscha) genome and transcriptome. *PLoS One* **13**(2018).

31. Aury, J.M. *et al.* Global trends of whole-genome duplications revealed by the ciliate *Paramecium tetraurelia*. *Nature* **444**, 171-8 (2006).

32. Arnaiz, O., Meyer, E. & Sperling, L. ParameciumDB 2019: integrating genomic data across the genus for functional and evolutionary biology. *Nucleic acids research* **48**, D599-D605 (2020).

33. Blanc-Mathieu, R. *et al.* Hybridization and polyploidy enable genomic plasticity without sex in the most devastating plant-parasitic nematodes. *PLoS Genetics* **13**, e1006777 (2017).

34. Venter, J.C. *et al.* The sequence of the human genome. *Science* **291**, 1304-1351 (2001).

35. Lander, E.S. *et al.* Initial sequencing and analysis of the human genome. *Nature* **409**, 860-921 (2001).

36. Braasch, I. *et al.* The spotted gar genome illuminates vertebrate evolution and facilitates human-teleost comparisons. *Nature Genetics* **48**, 427-37 (2016).

37. Goffeau, A. *et al.* Life with 6000 genes. *Science* **274**, 546,563-567 (1996).

38. Gordon, J.L., Byrne, K.P. & Wolfe, K.H. Additions, losses and rearrangements on the evolutionary route from a reconstructed ancestor to the modern *Saccharomyces cerevisiae* genome. *PLoS Genetics* **5**, e1000485 (2009).

39. Byrne, K.P. & Wolfe, K.H. Consistent patterns of rate asymmetry and gene loss indicate widespread neofunctionalization of yeast genes after whole-genome duplication. *Genetics* **175**, 1341-1350 (2007).

40. Cliften, P. *et al.* Finding functional features in *Saccharomyces* genomes by phylogenetic footprinting. *Science* **301**, 71-76 (2003).

41. Dujon, B. *et al.* Genome evolution in yeasts. *Nature* **430**, 35-44 (2004).

42. Gordon, J.L. *et al.* Evolutionary erosion of yeast sex chromosomes by mating-type switching accidents. *Proc Natl Acad Sci U S A* **108**, 20024-9 (2011).

43. Scannell, D.R. *et al.* Independent sorting-out of thousands of duplicated gene pairs in two yeast species descended from a whole-genome duplication. *Proceedings of the National Academy of Sciences, U.S.A.* **104**, 8397-8402 (2007).

44. Emery, M. *et al.* Preferential retention of genes from one parental genome after polyploidy illustrates the nature and scope of the genomic conflicts induced by hybridization. *PLoS Genetics* **14**, e1007267em (2018).

45. Huang, S. *et al.* The genome of the cucumber, Cucumis sativus L. *Nature genetics* **41**, 1275-1281 (2009).

46. Ming, R. *et al.* The pineapple genome and the evolution of CAM photosynthesis. *Nature genetics* **47**, 1435 (2015).

47. McGrath, C.L., Gout, J.-F., Doak, T.G., Yanagi, A. & Lynch, M. Insights into three whole-genome duplications gleaned from the Paramecium caudatum genome sequence. *Genetics* **197**, 1417-1428 (2014).

48. Stein, L., Sternberg, P., Durbin, R., Thierry-Mieg, J. & Spieth, J. WormBase: Network access to the genome and biology of *Caenorhabditis elegans*. *Nucleic Acids Research* **29**, 82-86 (2001).

49. Rondeau, E.B. *et al.* The genome and linkage map of the northern pike (Esox lucius): conserved synteny revealed between the salmonid sister group and the Neoteleostei. *PLoS One* **9**(2014).

50. Dehal, P. *et al.* The draft genome of Ciona intestinalis: insights into chordate and vertebrate origins. *Science* **298**, 2157-2167 (2002).

51. Conant, G.C. & Wagner, A. GenomeHistory: A software tool and its application to fully sequenced genomes. *Nucleic Acids Research* **30**, 3378-3386 (2002).

52. Altschul, S.F. *et al.* Gapped Blast and Psi-Blast : A new-generation of protein database search programs. *Nucleic Acids Research* **25**, 3389-3402 (1997).

53. Byrne, K.P. & Wolfe, K.H. The Yeast Gene Order Browser: Combining curated homology and syntenic context reveals gene fate in polyploid species. *Genome Research* **15**, 1456-1461 (2005).

54. Doring, A., Weese, D., Rausch, T. & Reinert, K. SeqAn an efficient, generic C++ library for sequence analysis. *BMC Bioinformatics* **9**, 11 (2008).
